# Gum arabic-OPO_3_H_2_ as a new natural-based green catalyst for the one-pot pseudo-four-component synthesis of naphtho[1,2-*e*][1,3]oxazines†

**DOI:** 10.1039/d0ra07199d

**Published:** 2020-11-06

**Authors:** Sahar Saadat Hosseinikhah, Bi Bi Fatemeh Mirjalili, Naeimeh Salehi, Abdolahamid Bamoniri

**Affiliations:** Department of Chemistry, College of Science, Yazd University P. O. Box 89195-741 Yazd Iran fmirjalili@yazd.ac.ir +983538210644 +983531232672; Department of Organic Chemistry, Faculty of Chemistry, University of Kashan Kashan Iran

## Abstract

Gum arabic-OPO_3_H_2_ (GA-OPO_3_H_2_) as a unique natural-based green catalyst was synthesized by the reaction of phosphorus pentoxide with gum arabic. The structure and properties of the catalyst were studied *via* several analysis methods such as FT-IR, MAPPING, EDS, SEM, XRD, and TGA. The efficiency of the above-mentioned catalyst was investigated for the synthesis of naphtho-1,3-oxazines *via* a pseudo-four-component reaction of primary amines, formaldehyde, and 2-naphthol under the solvent-free grinding condition at room temperature using an electrical mortar-heater. The obtained results indicated that GA-OPO_3_H_2_ is a highly efficient green catalyst for the synthesis of naphtho[1,2-*e*][1,3]oxazines with high yields, simple workup, and benign reaction condition.

## Abstract 2

Gum arabic-OPO_3_H_2_ (GA-OPO_3_H_2_) as a unique natural-based green catalyst was synthesized by the reaction of phosphorus pentoxide with gum arabic.

## Graphical abstract

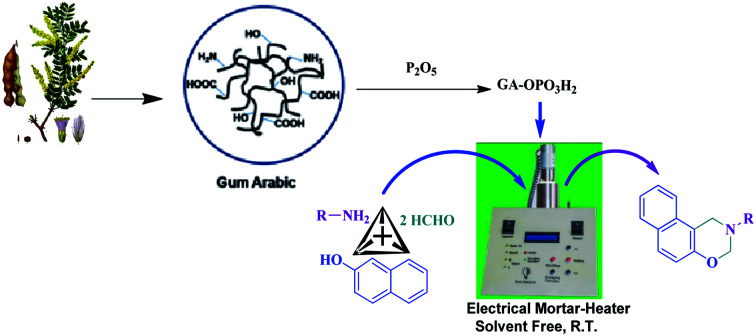

## Introduction

For the synthesis of organic compounds, the need for environmentally friendly conditions, green pathways, and low-cost with short reaction times is increasing. Therefore, it is preferable to use the natural-based catalysts and solvent-free methods. Gum Arabic (GA) is a biodegradable and renewable glucoprotein, which is an exudate collected from the stems and branches of Acacia trees.^1,2^ GA is also known to be a branched and complex acidic heteropolysaccharide whose main chain consists of (1→3)-β-d-galactopyranosyl units and side chains consists of l-arabinofuranosyl, l-rhamnopyranosyl, d-galactopyranosyl, and d-glucopyranosyluronic acid units.^3,4^ GA is generally safe, and in recent years, it has been widely used as a support,^5–9^ hydrogel^10,11^ and gold nanoparticle stabilizer.^12^ 1,3-Oxazines as an important class of heterocycles have wide biological and pharmacological activities such as antitumor,^13^ antibacterial,^14^ anti-HIV,^15^ analgesic,^16^ antihypertensive,^17^ antithrombotic,^18^ and antiulcer activities.^19^ Moreover, naphthoxazine derivatives have exhibited therapeutic potential for the treatment of Parkinson's disease.^20^

Based on the biological importance of benzo-fused 1,3-oxazines, numerous methods have been developed for the synthesis of pseudo-four-component including (1) Mannich-type condensation of 1- or 2-naphthol, formaldehyde, and a primary amine, (2) aza-acetalization of aromatic aldehydes with 2-(*N*-substituted aminomethyl) phenols in the presence of an acid as a catalyst and (3) electrooxidative cyclization of hydroxyamino compounds.^21^ The most common method for the synthesis of naphtho[1,2-*e*][1,3]oxazines is the multicomponent reaction of 2-naphthol, formaldehyde and a primary amine *via* a Mannich-type condensation. The synthesis of naphtho[1,2-*e*][1,3]oxazines using MCRs offers significant advantages including a reduction in the number of steps, energy consumption, and waste production.

Some of the catalysts that have previously been used for the synthesis of these products are KAl(SO_4_)_2_·12H_2_O (alum),^22^ ZrOCl_2_,^23^ 1-benzyl- 3-methyl imidazolium hydrogen sulfate [bnmim] [HSO_4_],^24^ polyethylene glycol (PEG),^25^ and thiamine hydrochloride (VB_1_).^26^ Despite the remarkable achievements for the synthesis of naphtho[1,2-*e*][1,3]oxazine derivatives, some of these catalysts have limitations such as inefficient separation of the catalyst from reaction mixtures, unrecyclable and environmental limitations. Furthermore, this reaction was performed without a catalyst under harsh reaction conditions.^27,28^

In this study, we wish to report an efficient eco-friendly procedure for the synthesis of naphtho[1,2-*e*][1,3]oxazines using GA-OPO_3_H_2_ as a new natural-based green catalyst *via* the reaction of primary amines, formaldehyde, and 2-naphthol.

## Results and discussion

**Scheme 1:**
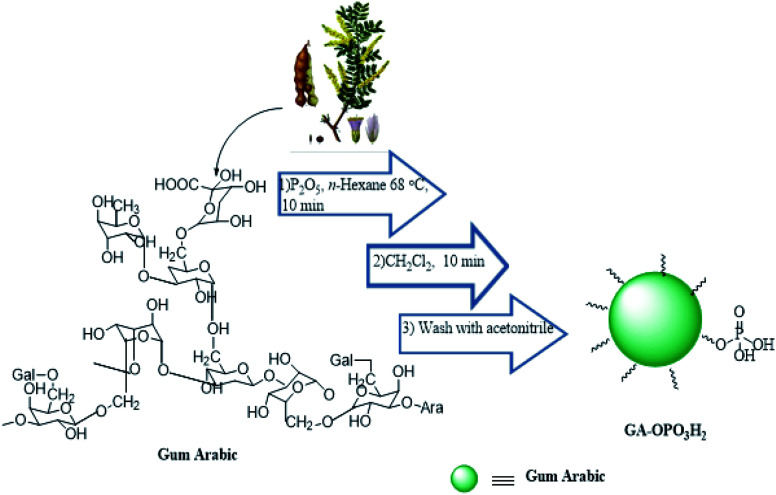
Preparation of GA-OPO_3_H.

In this study, an efficient and environmentally benign protocol was developed for the synthesis of naphtho[1,2-*e*][1,3]oxazine derivatives using a pseudo-four-component reaction of primary amines, formaldehyde, and 2-naphthol in the presence of GA-OPO_3_H_2_. The steps for the synthesis of the GA-OPO_3_H_2_ catalyst are shown in Scheme 1. The as-synthesized catalyst was characterized *via* FT-IR, XRD, SEM, MAPPING, EDS, and TGA.

**Fig. 1:**
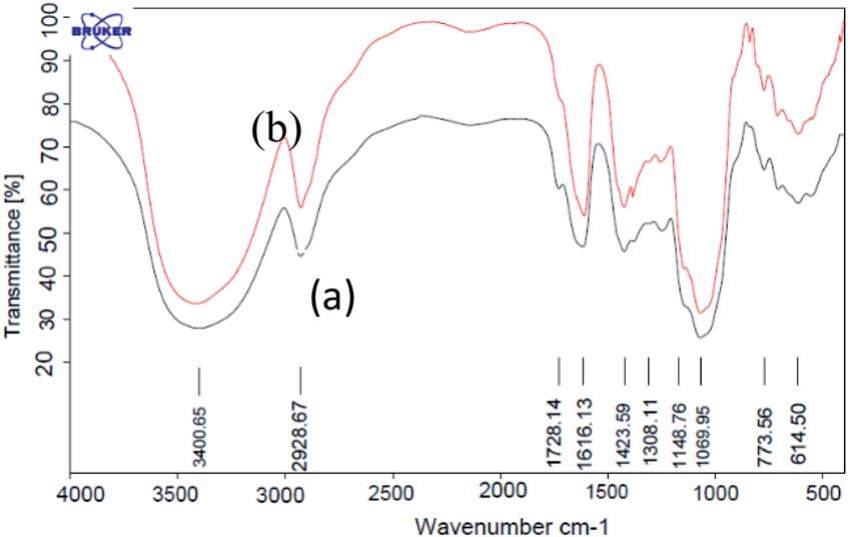
FT-IR spectra of (a) GA (b) GA-OPO_3_H_2_.

The FT-IR (KBR) spectra of GA and GA-OPO_3_H_2_ are shown in Fig. 1. The characteristic absorption bands of GA at 1069 and 1148 cm^−1^ (C–O, stretch), 1423 (O–H, bending), 1616 cm^−1^ (C=O, aliphatic acid), 2928 cm^−1^ (C–H, stretch), and 3000–3600 cm^−1^ (O–H, stretch) can be observed (Fig. 1a). The strong peaks in the range of 900–1200 cm^−1^ are the fingerprints of carbohydrates. These peaks are observed in the case of GA and GA-OPO_3_H_2_. The bands at 1071 cm^−1^ (P–O–C) and 1228 cm^−1^ (P=O) are attributed to the stretching vibrations of the phosphoric acid section that overlaps with the absorption bands of GA in this region.

**Fig. 2:**
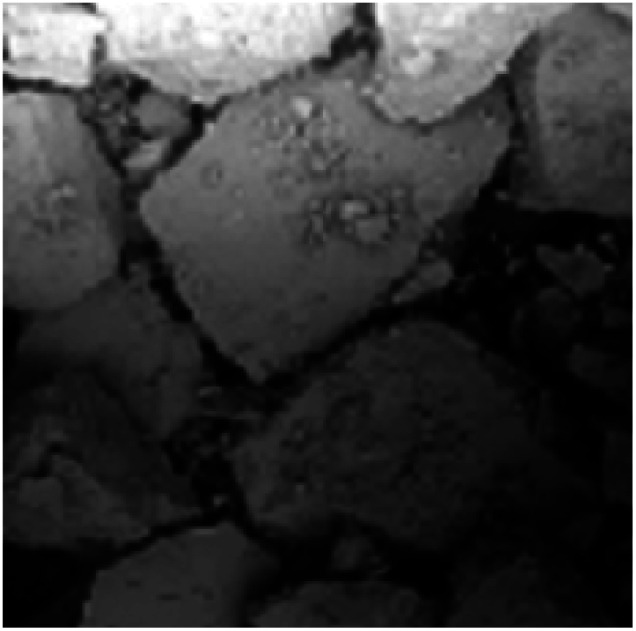
SEM image of GA-OPO_3_H_2_.

**Fig. 3:**
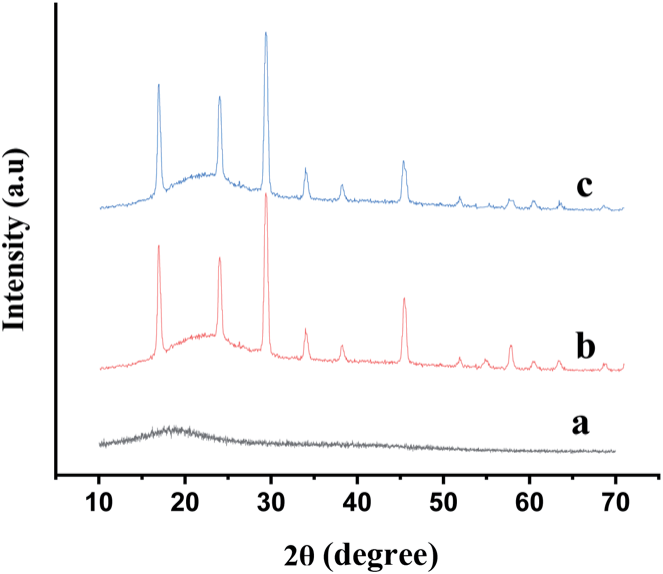
XRD patterns of the (a) GA, (b) GA-OPO_3_H_2_ and (c) reused GA-OPO_3_H_2_.

The particles size and morphology of GA-OPO_3_H_2_ were investigated *via* SEM. The exterior surface of GA-OPO_3_H_2_ appears as an irregular rocky surface in which the dimensions were found to be below 10 μm (Fig. 2). Fig. 3 illustrates the powder X-ray diffractograms obtained for natural GA, GA-OPO_3_H_2_, and reused GA-OPO_3_H_2_, respectively. The broad peak at 2*θ* = 19.895° (Fig. 3a) relates to the amorphous nature of GA. After the reaction of P_2_O_5_ with GA, the intensity of the corresponding peak decreases (Fig. 3b), verifying that P_2_O_5_ is bonded to GA. Other peaks in the GA-OPO_3_H_2_ spectrum may be due to the release of metals by the hydrolysis of calcium, magnesium, and potassium salts of GA, their binding to free hydroxyl groups, and the formation of metal complexes.

**Fig. 4:**
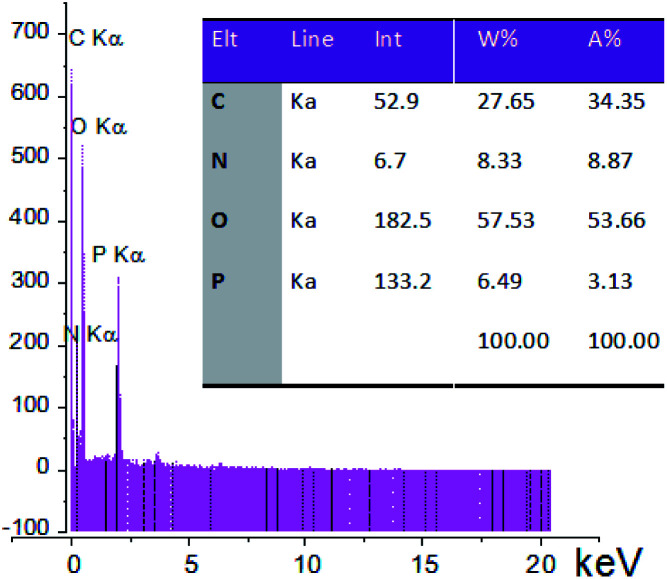
EDS spectra of GA-OPO_3_H_2_.

**Fig. 5:**
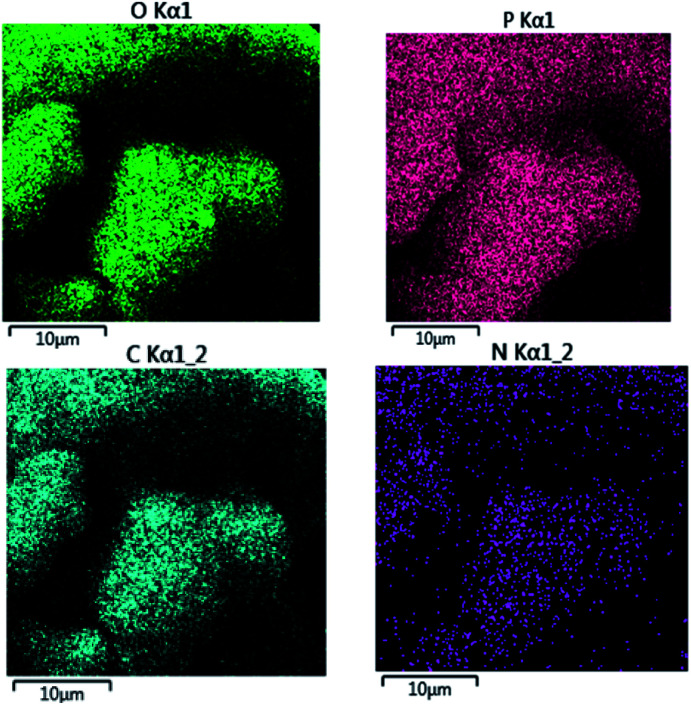
Elemental mapping images of GA-OPO_3_H_2_.

**Fig. 6:**
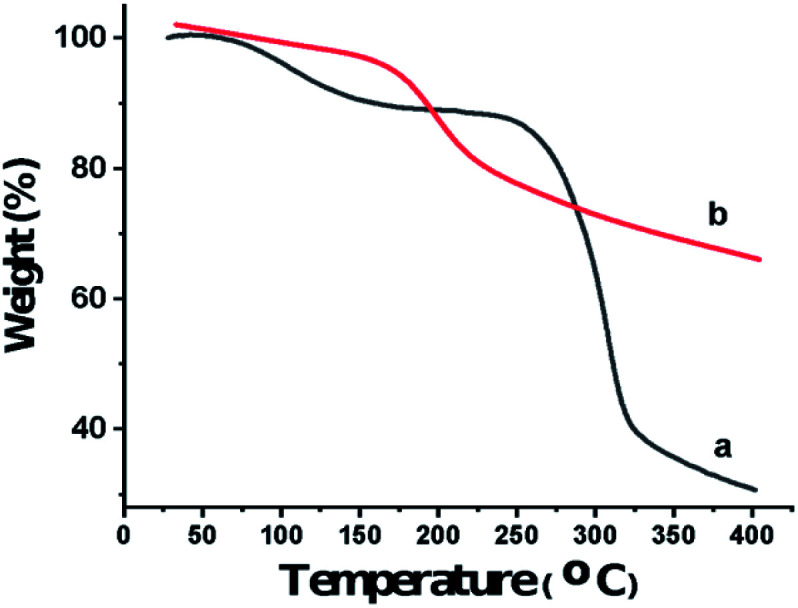
Thermal gravimetric analysis pattern of (a) gum arabic, (b) GA-OPO_3_H_2_.

Also, the presence of C, O, N, and P in the catalyst was investigated *via* EDX analysis data (Fig. 4). The percentages of O, C, N, and P in the catalyst are 57.53, 27.65, 8.33, and 6.49, respectively. Fig. 5 shows the elemental mapping of GA-OPO_3_H_2_. The images and patterns recorded confirm the presence of carbon, nitrogen, oxygen, and phosphorus elements in the catalyst. In addition, it shows that catalyst functional groups are well-scattered throughout the catalyst. The thermal stability of gum arabic and GA-OPO_3_H_2_ were evaluated *via* thermo-gravimetric analysis (TGA) in the temperature range of 33–404 °C (Fig. 6). The TGA curve of gum arabic shows three steps of weight loss: (a) 10% at 70–150 °C, (b) 50% at 250–320 °C and (c) 10% at 320–400 °C. The char yield of gum arabic at 400 °C is 30%. Also, the TGA curve of GA-OPO_3_H_2_ shows three steps weight loss which contains (a) 5 % at 30–120 °C, (b) 10% at 150–220 °C and (c) 15% at 250–400 °C. The first weight loss is attributed to the evaporation of free water and the second and third weight loss steps corresponded to decomposition and burning of the GA section of the catalyst. The char yield of GA-OPO_3_H_2_ at 400 °C is 63.96%. These evidences show that thermal stability of GA-OPO_3_H_2_ is higher than that of gum arabic. The acidic capacity of the catalyst was measured *via* titrating it with 0.03 N of NaOH. The number of OPO_3_H_2_ groups on the gum GA-OPO_3_H_2_ is 6.6 mmol g^−1^. The catalytic performance of GA-OPO_3_H_2_ was investigated in a one-pot reaction of 2-naphthol (1 mmol), formalin (2 mmol), and primary amine (1 mmol) towards the synthesis of naphtho[1,2-*e*][1,3]oxazines. Initially, the optimization experiments were performed in the reaction of 2-naphthol (1 mmol), formalin (2 mmol), aniline (1 mmol)) as the model reaction. The reaction was optimized *via* various parameters such as temperature, solvent, and catalyst amounts, and the results are summarized in Table 1. To investigate the effect of reaction temperature, the reaction was performed at different temperatures under solvent-free conditions (Table 1, entries 8–11).

**Table 1:** The reaction of 2-naphthol (1 mmol), formalin (2 mmol) and aniline (1 mmol) under various conditions

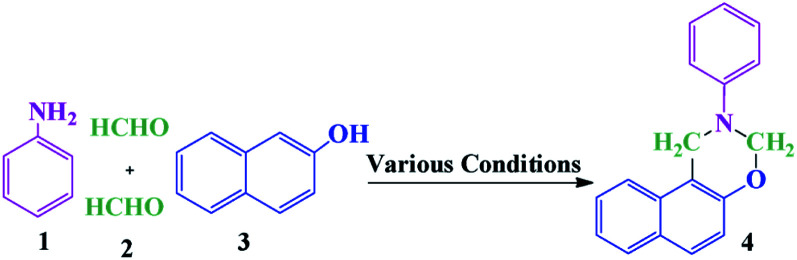					
Entry	Solvent	Catalyst^a^ (g [mmol])	Condition	Time (min)	Yield^b^ (%)
1	—	—	r. t.^c^	60	15
2	H_2_O	0.05 [0.33]	r. t.	25	56
3	C_2_H_5_OH	0.05 [0.33]	r. t.	25	69
4	CHCl_3_	0.05 [0.33]	r. t.	25	49
5	CH_3_CN	0.05 [0.33]	r. t.	25	62
6	H_2_O : C_2_H_5_OH (1 : 1)	0.05 [0.33]	r. t.	25	63
7	CH_3_OH	0.05 [0.33]	r. t.^c^	25	55
8	—	0.05 [0.33]	r. t.^c^	5	94
9	—	0.05 [0.33]	50 °C^c^	60	25
10	—	0.05 [0.33]	60 °C^c^	60	17
11	—	0.05 [0.33]	80 °C^c^	60	—
12	—	0.04 [0.26]	r. t. c	5	99
13	—	0.03 [0.2]	r. t. c	5	92
14	—	0.02 [0.13]	r. t. c	5	87

a GA-OPO_3_H_2_.

b Isolated yield.

c Electrical mortar-heater.

The highest yield was achieved at room temperature using an electrical mortar-heater (Table 1, entry 8). Different solvents including H_2_O, EtOH, CHCl_3_, CH_3_CN, H_2_O : EtOH, MeOH were also screened (Table 1, entries 2–7). The model reaction was easier and gave the highest yield in a solvent-free condition. To optimize the catalyst amount, the model reaction was carried out in the presence of various amounts of the catalyst, and according to the obtained results, the optimum amount of the catalyst was 0.04 g (Table 1, entry 12). In a reaction, without catalyst, a low yield of the product was achieved after a long reaction time (Table 1, entry 1), and this indicates the high efficiency of catalyst for this reaction. According to the results, the best condition is using 0.04 g of the catalyst under the solvent-free condition at room temperature with an electrical mortar-heater (Table 1, entry 12).

Due to remarkable results from the above experiments, we decided to synthesize naphtho[1,2-*e*][1,3]oxazines, and the results are summarized in Table 2. The products were obtained in good to excellent yields in short reaction times with aliphatic and aromatic amines with electron-withdrawing/donating substituents.

**Table 2:** Synthesis of naphtho[1,2-*e*][1,3]oxazines in the presence of GA-OPO_3_H_2_ under solvent-free condition at room temperature^a^

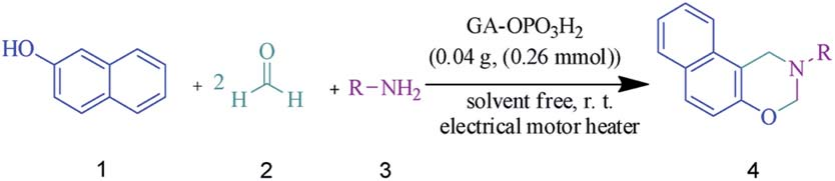					
Entry	R	Product	Time (min)	Yield^b^ (%)	MP (°C)
1	C_6_H_5_–	4a	9	99	45–47 (ref. 22)
2	4-Me–C_6_H_4_–	4b	10	94	86–88 (ref. 22)
3	4-Et–C_6_H_4_–	4c	10	91	44–46 (ref. 21)
4	4-Br–C_6_H_4_–	4d	9	97	116–118 (ref. 22)
5	4-Cl–C_6_H_4_–	4e	9	97	100–104 (ref. 21)
6	4-OMe–C_6_H_4_–	4f	9	95	76–78 (ref. 22)
7	C_6_H_5_–CH_2_-	4g	10	91	124–125 (ref. 21)
8	2-Cl–C_6_H_4_–CH_2_-	4h	11	90	72–73 (ref. 21)
9	C_6_H_5_–CH_2_–CH_2_-	4i	9	97	233(d) (ref. 21)
10	2-Furyl–CH_2_–	4j	9	94	98–100 (ref. 21)
11	Cyclohexyl-	4k	11	92	250(d)^c^ (ref. 21)
12	*n*-Butyl–	4l	11	94	170(d)^c^ (ref. 21)
13	*n*-Hexyl–	4m	11	95	178(d)^c^ (ref. 21)

a The amount ratio of 1 (mmol) : 2 (mmol) : 3 (mmol): GA-OPO_3_H_2_ (mmol) is equal to 1 : 2 : 1 : 0.26.

b Isolated yield.

c Decompose.

**Fig. 7:**
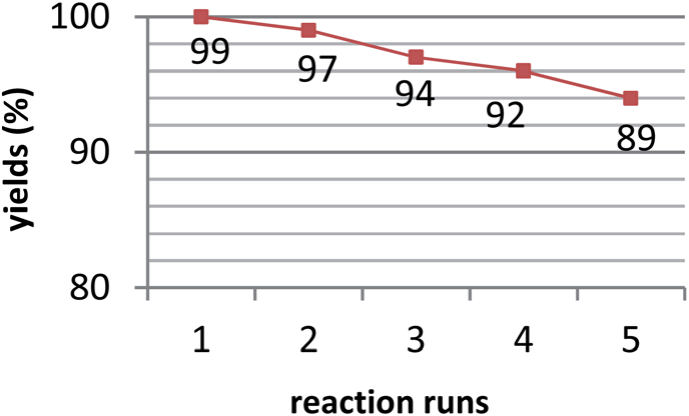
Catalyst recycling experiments.

After the completion of the reaction, the reusability of the catalyst was also investigated in the model reaction. It was separated by filtration, washed 3 times with ethanol, and dried in an oven at 50 °C to provide an opportunity for recycling experiments. The separated catalyst was reused in the mentioned reaction five times without considerable loss of its catalytic activity (Fig. 7).

The separated catalyst was reused in the mentioned reaction five times without considerable loss of its catalytic activity (Fig. 7 and 3c).

The efficiency of the GA-OPO_3_H_2_ catalyst in comparison with some previously reported ones for the synthesis naphtho[1,2-*e*][1,3]oxazines are summarized in Table 3. As Table 3 indicates, the use of GA-OPO_3_H_2_ has remarkably improved the synthesis of naphtho[1,2-*e*][1,3]oxazines in different terms such as simplicity of protocol with compatibility with the environment.

**Table 3:** Comparison of the efficiency of the GA-OPO_3_H_2_ catalyst with other reported catalysts for the synthesis of naphtho[1,2-*e*][1,3]oxazines (4a)

Entry	Conditions	Time (min)	Yield^d^ (%)
	Solvent/temp(°C)/catalyst		
1	H_2_O/r.t.^a^/KAl(SO_4_)_2_·12H_2_O	15	75 (ref. 22)
2	—/r.t/ZrOCl_2_	0.5	80 (ref. 23)
3	—/r.t/[bnmim][HSO_4_]^b^	1	77 (ref. 24)
4	—/r.t/(PEG)^c^	5	89 (ref. 27)
5	H_2_O/r.t/thiamine hydrochloride (VB_1_)	60	65 (ref. 26)
6	H_2_O/r.t/nano-Al_2_O_3_/BF_3_/Fe_3_O_4_	20	92 (ref. 29)
7	—/r.t./GA-OPO_3_H_2_	9	99 (this work)

a Room temperature.

b 1-Benzyl-3-methyl imidazolium hydrogen sulphate.

c Polyethylene glycol.

d Isolated yield.

**Scheme 2:**
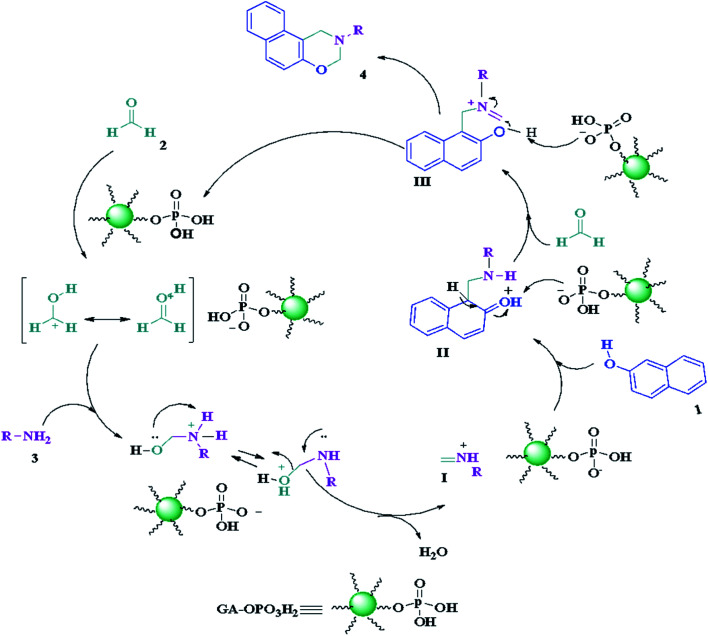
Proposed mechanism for the synthesis of naphtho[1,2-*e*][1,3]oxazines.

The suggested mechanism for the synthesis of naphtho[1,2-*e*][1,3]oxazines has been shown in Scheme 2. The GA-OPO_3_H_2_ catalyst operates as a Brønsted acid and at first, actuates the carbonyl group in formaldehyde. Then, the Mannich-type condensation of amine (3) with formaldehyde (2) gives imine I, which was attacked by the electron-rich center of 2-naphthol (1) to form intermediate II. Intermediate II, *via* a second Mannich-type condensation with a second molecule of formaldehyde, gives intermediate III, which through intramolecular cyclization afforded product (4).

## Conclusions

In conclusion, we have introduced GA-OPO_3_H_2_ as a highly efficient, eco-friendly, inexpensive, and natural-based heterogeneous catalyst. The invented catalyst conveniently catalyzed the synthesis of naphtho[1,2-*e*][1,3]oxazine derivatives *via* a one-pot three-component reaction of 2-naphthol, formalin, and aniline using GA-OPO_3_H_2_ under the solvent-free condition at room temperature with an electrical mortar-heater. Short reaction time and excellent yields are among the advantages of this novel methodology.

## Experimental

### Materials and methods

All compounds were purchased from Merck, Aldrich, and Fluka chemical companies and used without any additional purification. The electrical mortar-heater that was used for the grinding of the reaction mixture, was purchased from Borna-Kherad Co., Iran, Yazd. FT-IR spectra were recorded on a Bruker, Equinox 55 spectrometer. A Bruker (DRX-400 Avanes) NMR was used to record the ^1^H-NMR and ^13^C-NMR spectra. Melting points were determined by a Buchi melting point B-540 B. V. CHI apparatus and were uncorrected. X-ray diffraction (XRD) patterns were obtained by a Philips Xpert MPD diffractometer equipped with a Cu Kα anode (*k* = 1.54 Å) in the 2*θ* range from 10° to 80°. Quantitative elemental information and maps of GA-OPO_3_H_2_ were studied *via* energy-dispersive X-ray spectroscopy (EDS) by a Phenom pro X instrument. Thermal gravimetric analysis (TGA) was conducted using a “Universal V4.5A TA” instrument. The morphology was studied using a Philips XL30 scanning electron microscope (SEM).

### Preparation of GA-OPO_3_H_2_

GA is an exudate from the Acacia tree. Then, the catalyst (GA-OPO_3_H_2_) is simply prepared by adding P_2_O_5_ (3 g) in the *n*-hexane solution (5 ml) containing gum arabic (1 g). The mixture was stirred for 10 min at 68 °C. The mixture is sticky; hence, we have added dichloromethane (5 ml) to make it into a powder. The resulted suspension was filtered and washed 2 times with acetonitrile to remove unreacted P_2_O_5_ and dried at room temperature.

### General procedure the synthesis of naphtho[1,2-*e*][1,3]oxazines

A mixture of 2-naphthol (1 mmol), formalin (2 mmol), primary amine (1 mmol), and GA-OPO_3_H_2_ (0.04 g) was stirred under the solvent-free condition at room temperature with an electrical mortar-heater. After the completion of the reaction (monitored by TLC, *n*-hexane: EtOAc (4 : 1)), the reaction mixture was dissolved in hot ethanol (4 ml), and the catalyst was separated *via* filtration. After the evaporation of the solvent, the crude products were recrystallized from ethanol to give pure naphtho-1,3-oxazine derivatives. The recovered catalyst was washed 3 times with ethanol, dried in an oven at 50 °C, and then reused successfully.

## Conflicts of interest

There are no conflicts to declare.

## Supplementary Material

RA-010-D0RA07199D-s001

## References

[cit1] Mao P., Zhao M., Zhang F., Fang Y., Phillips G. O., Nishinari K., Jiang F. (2013). Carbohydr. Polym..

[cit2] Fazeli-Attar S. A., Mirjalili B. F. (2018). Environ. Chem. Lett..

[cit3] Gils P. S., Ray D., Sahoo P. K. (2010). Int. J. Biol. Macromol..

[cit4] Ling M., Zhao H., Xiaoc X., Shi F., Wu M., Qiu J., Li S., Song X., Liu G., Zhang S. (2015). J. Mater. Chem. A.

[cit5] Khazaei A., Rahmati S., Ghaderi A., Roshani L. (2014). J. Iran. Chem. Soc..

[cit6] Baran T., Menteş A. (2020). Int. J. Biol. Macromol..

[cit7] Sreedhar B., Surendra Reddy P., Keerthi Devi D. (2009). J. Org. Chem..

[cit8] Wu C., Chen D. (2012). Nanoscale Res. Lett..

[cit9] Sreedhar B., Reddy P. S., Devi D. K. (2009). J. Org. Chem..

[cit10] Favaro S. L., Oliveira F. D., Reis A. V., Guilherme M. R., Muniz E. C., Tambourgi E. B. (2008). J. Appl. Polym. Sci..

[cit11] Paulino A. T., Guilherme M. R., Mattoso L. H. C., Tambourgi E. B. (2010). Macromol. Chem. Phys..

[cit12] Kattumuri V., KattiK Bhaskaran S., Boote E. J., Casteel S. W., Fent G. M., Robertson D. J., Chandrasekhar M., Kannan R., Katti K. V. (2007). Nano.Micro.Small.

[cit13] Chylińska J., Urbański T., Mordarski M. (1963). J. Med. Chem..

[cit14] Mathew B. P., Kumar A., Sharma S., Shukla P. K., Nath M. (2010). Eur. J. Med. Chem..

[cit15] Cocuzza A. J., Chidester D. R., Cordova B. C., Jeffrey S., Parsons R. L., Bacheler L. T., Erickson-Viitanen S., Trainor G. L., Ko S. S. (2001). Bioorg. Med. Chem. Lett..

[cit16] Kurz T. (2005). Tetrahedron.

[cit17] Kajino M., Shibouta Y., Nishikawa K., Meguro K. (1991). Chem. Pharm. Bull..

[cit18] Buckman B. O., Mohan R., Koovakkat S., Liang A., Trinh L., Morrissey M. M. (1998). Bioorg. Med. Chem. Lett..

[cit19] Katsura Y., Nishino S., Takasugi H. (1991). Chem. Pharm. Bull..

[cit20] Joyce J. N., Presgraves S., Renish L., Borwege S., Osredkar T., Hagner D., Replogle M., PazSoldan M., Millan M. J. (2003). Exp. Neurol..

[cit21] Azad S., Mirjalili B. F. (2019). Mol. Diversity.

[cit22] Sadaphal S. A., Sonar S. S., Shingate B. B., Shingare M. S. (2010). Green Chem. Lett. Rev..

[cit23] Kategaonkar A. H., Sonar S. S., Pokalwar R. U., Kategaonkar A. H., Shingate B. B., Shingare M. S. (2010). Bull. Korean Chem. Soc..

[cit24] Kategaonkar A. H., Sonar S. S., Shelke K. F., Shingate B. B., Shingare M. S. (2010). Org. Commun..

[cit25] Shinde P. V., Kategaonkar A. H., Shingate B. B., Shingare M. S. (2011). Chin. Chem. Lett..

[cit26] Dhakane V. D., Gholap S. S., Deshmukh U. P., Chavan H. V., Bandgar B. P. (2014). C. R. Chim..

[cit27] Shen A. Y., Tsai C. T., Chen C. L. (1999). Eur. J. Med. Chem..

[cit28] Woodgate P. D., Horner G. M., Maynard N. P., Rickard C. E. (1999). J. Organomet. Chem..

[cit29] Babaei E., Mirjalili B. F. (2019). Polycyclic Aromat. Compd..

