# Supplementary material for: Gum arabic-OPO3H2 as a new natural-based green catalyst for the one-pot pseudo-four-component synthesis of naphtho[1,2-e][1,3]oxazines
Source: RSC Adv. 2020 Nov 6;10(66):40508–13. doi: 10.1039/d0ra07199d (PMC9057564; doi:10.1039/d0ra07199d)
Supplement: RA-010-D0RA07199D-s001 [file RA-010-D0RA07199D-s001.pdf]

Electronic Supplementary Material (ESI) for RSC Advances.
This journal is © The Royal Society of Chemistry 2020

Gum Arabic-OPO_3H_2 as a new natural-based green catalyst for one-pot *pseudo*-four-component synthesis of naphtho[1,2-*e*][1,3]oxazines

Sahar Saadat Hosseinikhah,[a] Bi Bi Fatemeh Mirjalili,*[a] Naeimeh Salehi,[a] Abdolahamid Bamoniri[b]

[a] *Department of Chemistry, College of Science, Yazd University, Yazd, P.O.Box 89195-741, Iran. E-mail: fmirjalili@yazd.ac.ir. Tel: +983531232672. Fax: +983538210644.*

[b] *Department of Organic Chemistry, Faculty of Chemistry, University of Kashan, Kashan, I.R.IRAN*

2-Phenyl-2,3-dihydro-1*H*-naphtho[1,2-*e*][1,3]oxazine (4a) ***(Table 2, entry 1):***

White solid, m.p. 45-47 ˚C; FT-IR (ATR) ῡ (cm^{-1}): 3060, 1600, 1496, 1231, 1057; ^{1}H NMR (Acetone-d_6, 400 MHz) /δ ppm: 7.89 (d, 1H, ^{3}J= 8.4 Hz, Ar-H), 7.85 (d, 1H, ^{3}J= 8 Hz, Ar-H), 7.73 (d, 1H, ^{3}J= 9.2 Hz, Ar-H), 7.53-7.57 (m, 1H, Ar-H), 7.38-7.42 (m, 1H, Ar-H), 7.23-7.28 (m, 4H, Ar-H), 7.04 (d, 1H, ^{3}J= 8.8 Hz, Ar-H), 6.87-6.91 (m, 1H, Ar-H), 5.54 (s, 2H, O-CH_2-N), 5.06 (s, 2H, -Ar-CH_2-N).

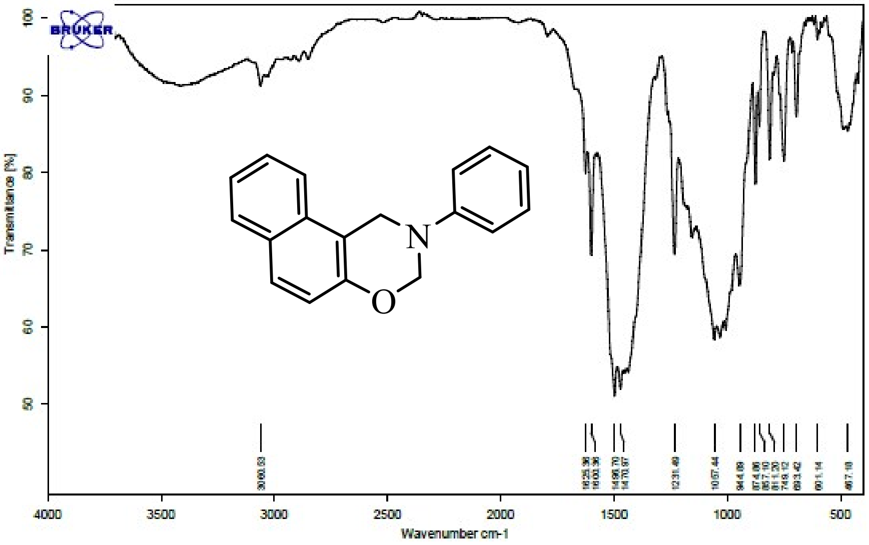

The FT-IR spectrum of product (**4a**)

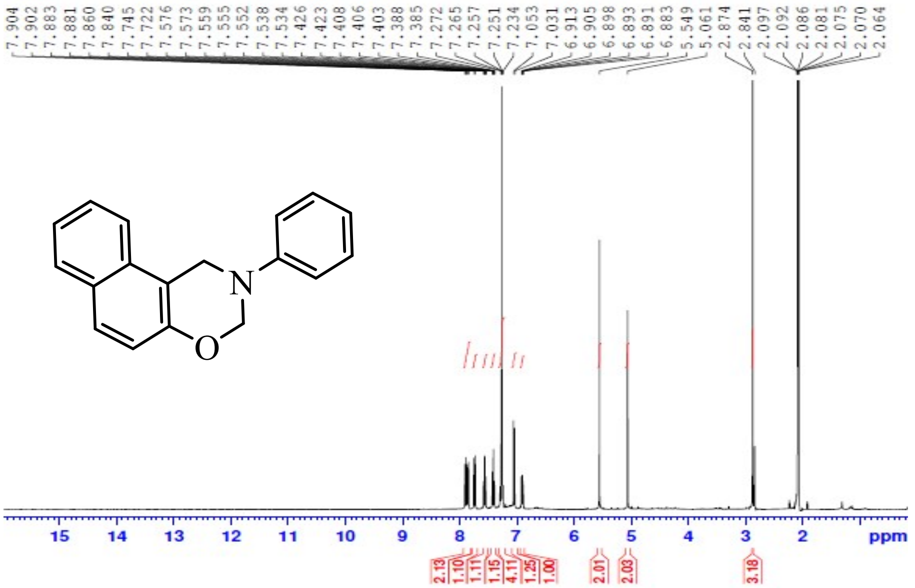

The ^{1}H NMR (400 MHz) spectrum of product (**4a**)

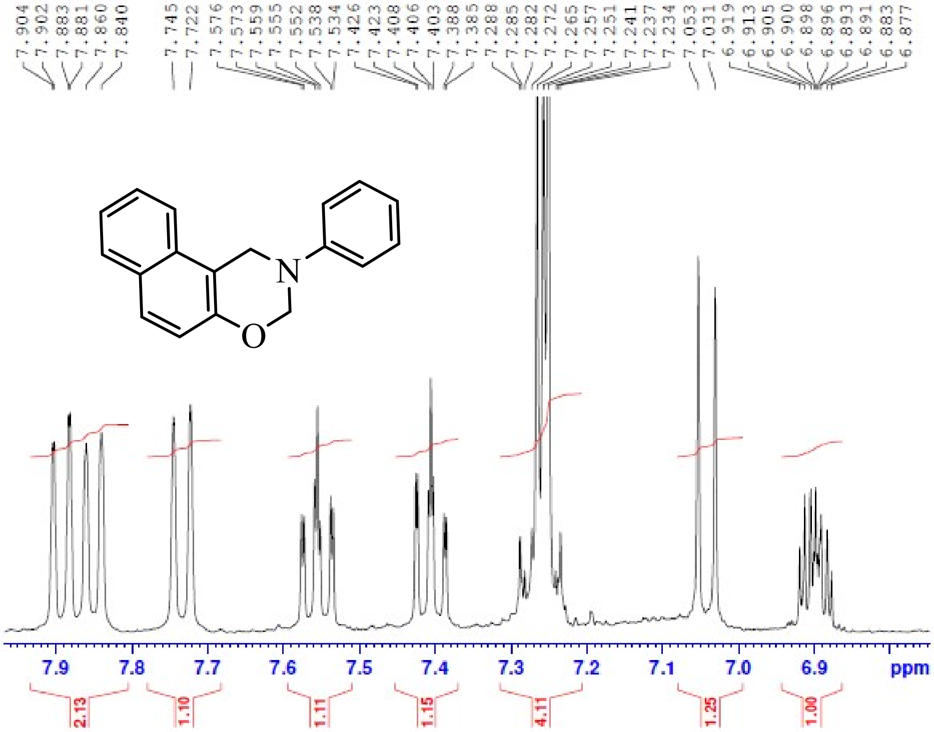

The ^{1}H NMR (400 MHz) spectrum of product (**4a**)

2,3-Dihydro-1*H*-naphtho[1,2-*e*][1,3]oxazine(4b) ***(Table 2, entry 2):***

Yellow solid, m.p. 87-89 ˚C; FT-IR (ATR) ῡ (cm^{-1}): 1597, 1470, 1226, 1096; ^{1}H NMR (Acetone-d_6, 400 MHz) /δ ppm: 7.82 (t, 2H, ^{3}J= 8.8 Hz, Ar-H), 7.69 (d, 1H, ^{3}J= 9.2 Hz, Ar-H), 7.51 (t, 1H, ^{3}J= 8 Hz, Ar-H), 7.36 (t, 1H, ^{3}J= 8 Hz, Ar-H), 6.99-7.04 (m, 3H, Ar-H), 7.09-7.11 (m, 2H, Ar-H), 5.52 (s, 2H, O-CH_2-N), 5.02 (s, 2H, -Ar-CH_2-N), 2.24 (s, 3H, CH_3-Ar).

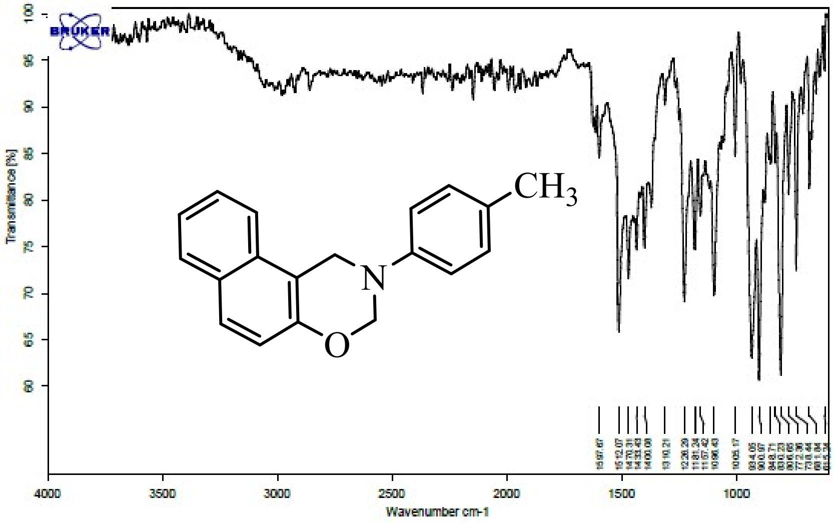

The FT-IR spectrum of product (**4b**)

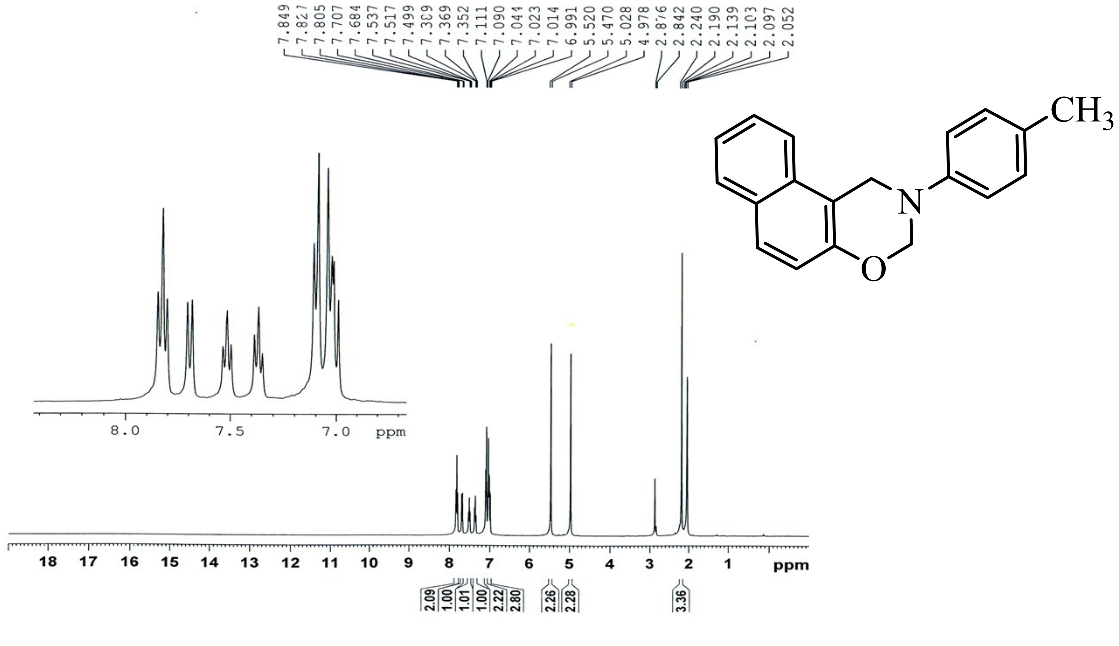

The ^{1}H NMR (400 MHz) spectrum of product (**4b**)

2-(4-Ethylphenyl)-2,3-dihydro-1*H*-naphtho[1,2-*e*][1,3]oxazine (4c) ***(Table 2, entry 3):***

Off-white solid, m.p. 44-46 °C; FT-IR (ATR) ῡ (cm^{-1}): 1597, 1467, 1226, 1056; ^{1}H NMR (Acetone-d_6, 400 MHz) /δ ppm: 7.80-7.85 (m, 2H, Ar-H), 7.69-7.71 (m, 1H, Ar-H), 7.50-7.55 (m, 1H, Ar-H), 7.35-7.40 (m, 1H, Ar-H), 6.99-7.14 (m, 5H, Ar-H), 5.49 (s, 2H, O-CH_2-N), 5.00 (s, 2H, -Ar-CH_2-N), 2.49-2.54 (m, 2H, -<u>CH_2</u>-CH_3), 1.15 (t, 3H, ^{3}J= 7 Hz, -CH_2-<u>CH_3</u>).

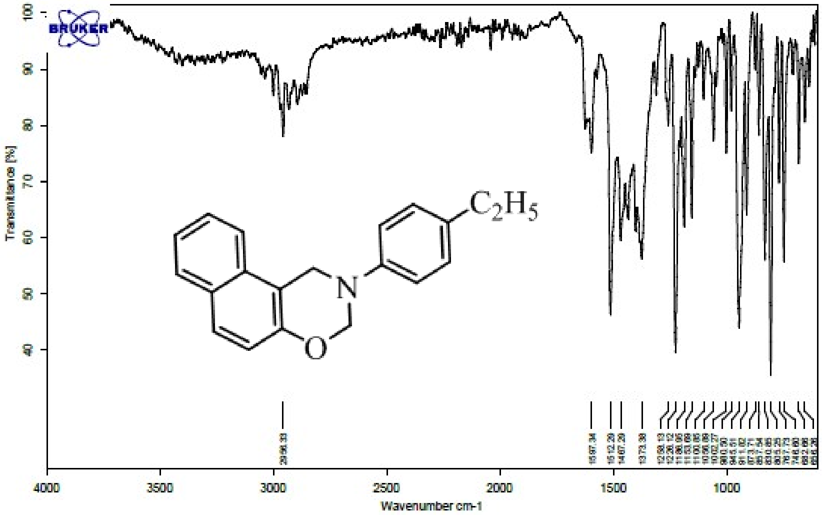

The FT-IR spectrum of product (**4c**)

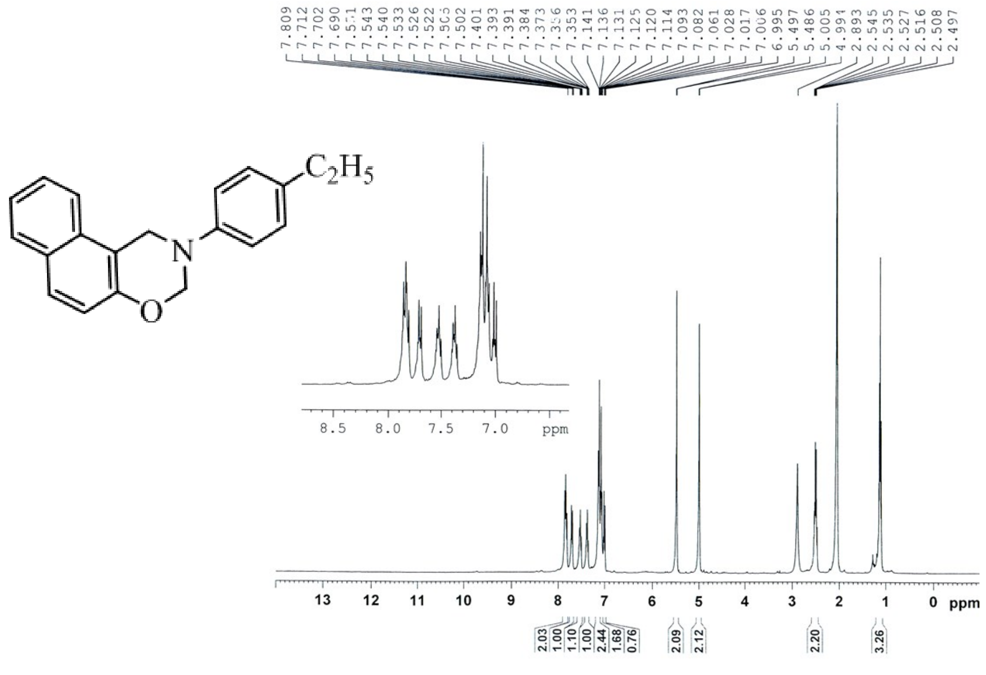

The ^{1}H NMR (400 MHz) spectrum of product (**4c**)

2-(4-Bromophenyl)-2,3-dihydro-1*H*-naphtho[1,2-*e*][1,3]oxazine(4d) ***(Table 2, entry 4):***

White solid, m.p. 116-119 ˚C; FT-IR (ATR) ῡ (cm^{-1}): 1588, 1489, 1223, 1092; ^{1}H NMR (Acetone-d_6, 400 MHz) /δ ppm: 7.84 (t, 2H, ^{3}J= 9.2 Hz, Ar-H), 7.71 (d, 1H, ^{3}J= 9.2 Hz, Ar-H), 7.52 (t, 1H, ^{3}J= 8.4 Hz, Ar-H), 7.37-7.40 (m, 3H, Ar-H), 7.20 (d, 2H, ^{3}J= 9.2 Hz, Ar-H), 7.01 (d, 1H, ^{3}J= 9.2 Hz, Ar-H), 5.51 (s, 2H, O-CH_2-N), 5.03(s, 2H, -Ar-CH_2-N).

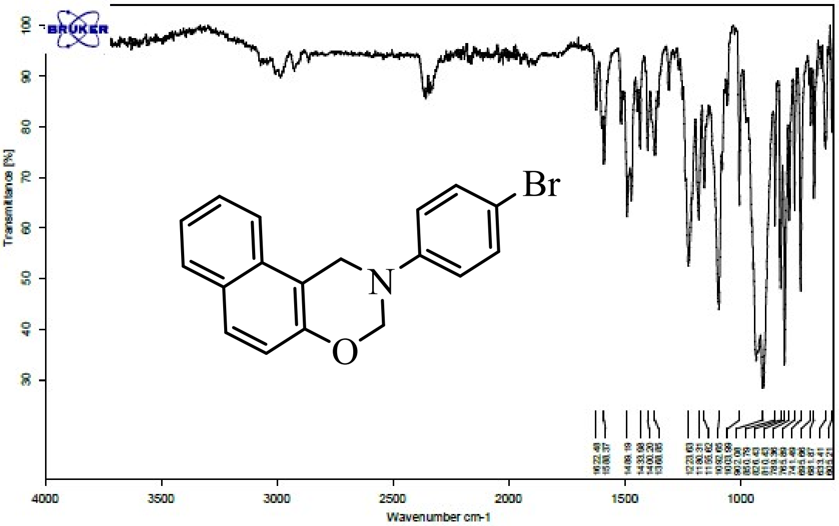

The FT-IR spectrum of product (**4d**)

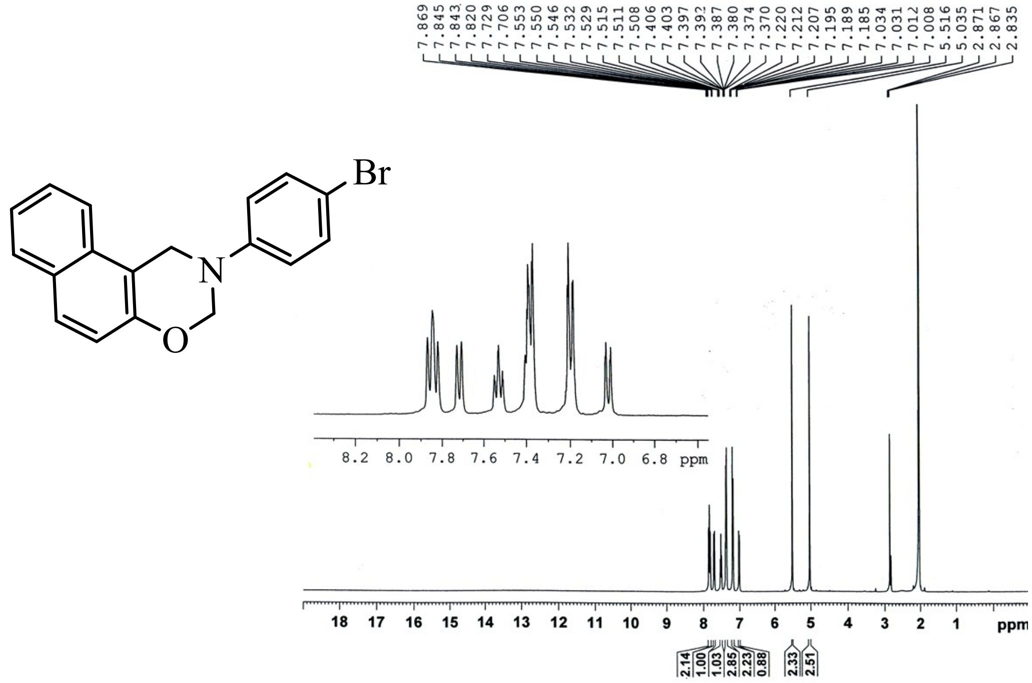

The ^{1}H NMR (400 MHz) spectrum of product (**4d)**

2-(4-Chlorophenyl)-2,3-dihydro-1*H*-naphtho[1,2-*e*][1,3]oxazine(4e) ***(Table 2, entry 5):***

White solid, m.p. 100-103 °C; FT-IR (ATR) ῡ (cm^{-1}): 1593, 1492, 1225, 1092; ^{1}H NMR (Acetone-d_6, 400 MHz) /δ ppm: 7.84 (t, 2H, ^{3}J= 9.2 Hz, Ar-H), 7.71 (d, 1H, ^{3}J= 8.8 Hz, Ar-H), 7.53 (t, 1H, ^{3}J= 7.2 Hz, Ar-H), 7.38 (t, 1H, ^{3}J= 7.6 Hz, Ar-H), 7.24-7.29 (m, 4H, Ar-H), 7.02 (d, 1H, ^{3}J= 8.8 Hz, Ar-H), 5.51 (s, 2H, O-CH_2-N), 5.03(s, 2H, -Ar-CH_2-N).

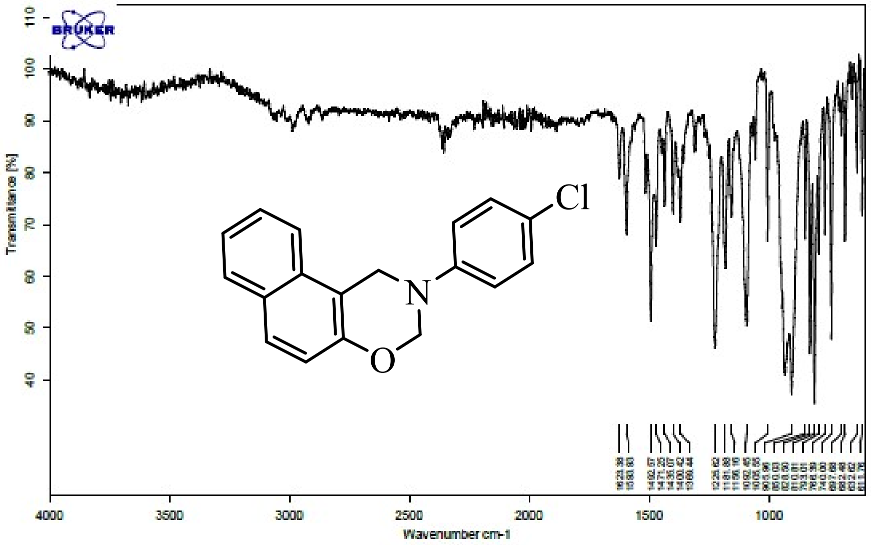

The FT-IR spectrum of product (**4e**)

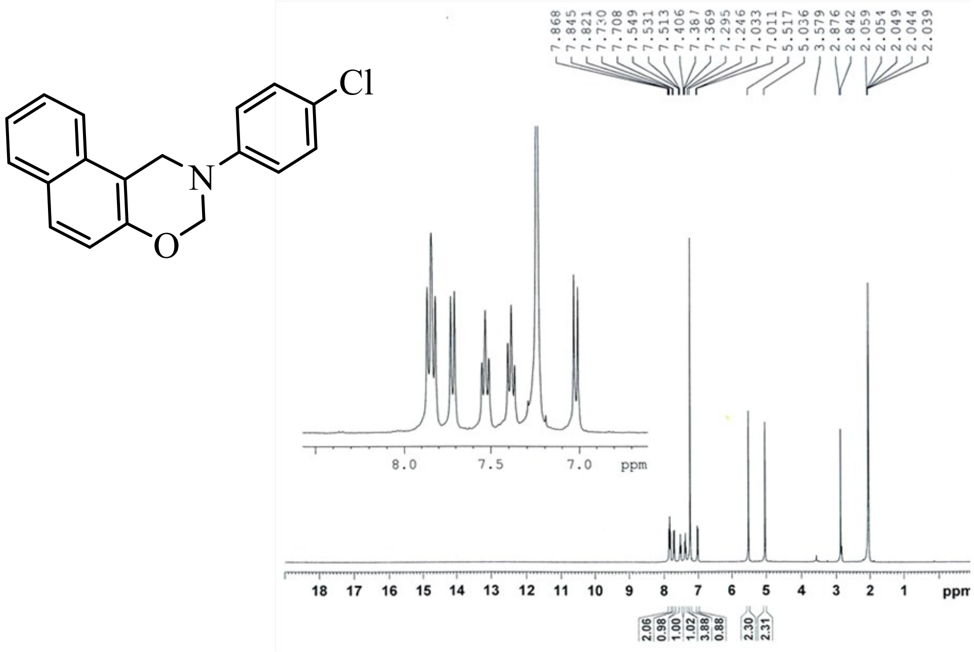

The ^{1}H NMR (400 MHz) spectrum of product (**4e)**

2-(4-Methoxyphenyl)-2,3-dihydro-1*H*-naphtho[1,2-*e*][1,3]oxazine(4f) ***(Table 2, entry 6):***

Off-white solid, m.p. 75-77 °C; FT-IR (ATR) ῡ (cm^{-1}): 1596, 1467, 1246, 1230, 1031; ^1H NMR (Acetone-d_6, 400 MHz) /δ ppm: 7.80-7.82 (m, 2H, Ar-H), 7.70 (d, 1H, ^3J= 8.8 Hz, Ar-H), 7.51 (m, 1H, Ar-H), 7.36 (m, 1H, Ar-H), 7.13 (d, 2H, ^3J= 7.2 Hz, Ar-H), 7.00 (d, 1H, ^3J= 8.8 Hz, Ar-H), 6.80 (d, 2H, ^3J= 7.2 Hz, Ar-H), 5.42 (s, 2H, O-CH_2-N), 4.93(s, 2H, -Ar-CH_2-N), 3.68 (s, 3H, O-CH_3).

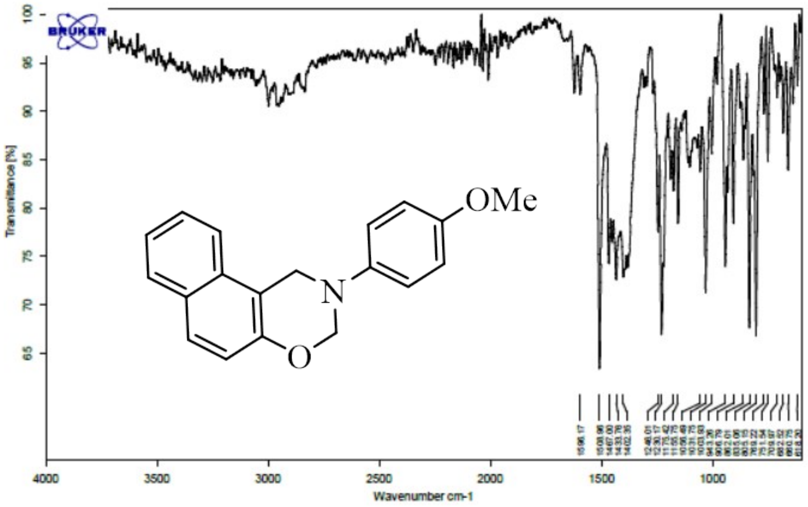

The FT-IR spectrum of product (**4f**)

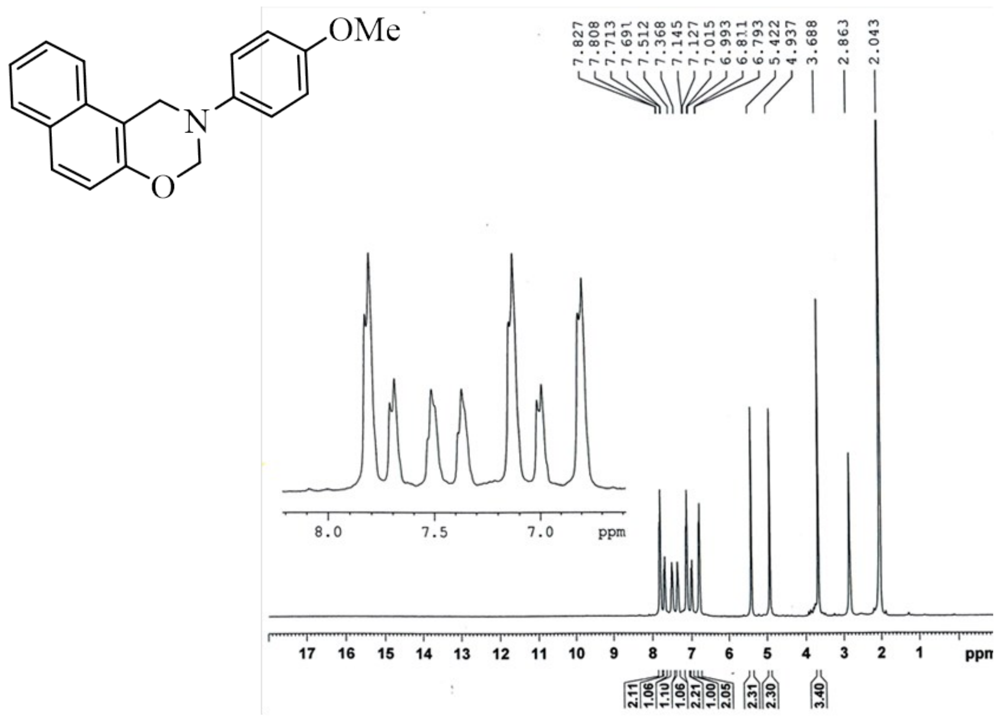

The ^1H NMR (400 MHz) spectrum of product (**4f**)

2-Benzyl-2,3-dihydro-1*H*-naphtho[1,2-*e*][1,3]oxazine(4g) ***(Table 2, entry 7):***

Off-white, m.p. 123-125 °C; FT-IR (ATR) ῡ (cm^{-1}): 1623, 1597, 1461, 1223, 1058, 738.
^{1}H NMR (DMSO-d_6, 500 MHz) /δ ppm: 7.05-7.71 (m, 11H, Ar-H), 4.89 (s, 2H, O-CH_2-N), 4.22 (s, 2H, -Ar-CH_2-N), 3.89 (s, 2H, -Ar-CH_2-N); ^{13}C NMR (DMSO-d_6, 100 MHz) /δ ppm: 46.55, 55.17, 81.55, 111.67, 118.32, 121.15, 123.37, 126.57, 127.19, 127.76, 128.34, 128.39, 128.49, 128.59, 131.53, 138.33, 151.41.

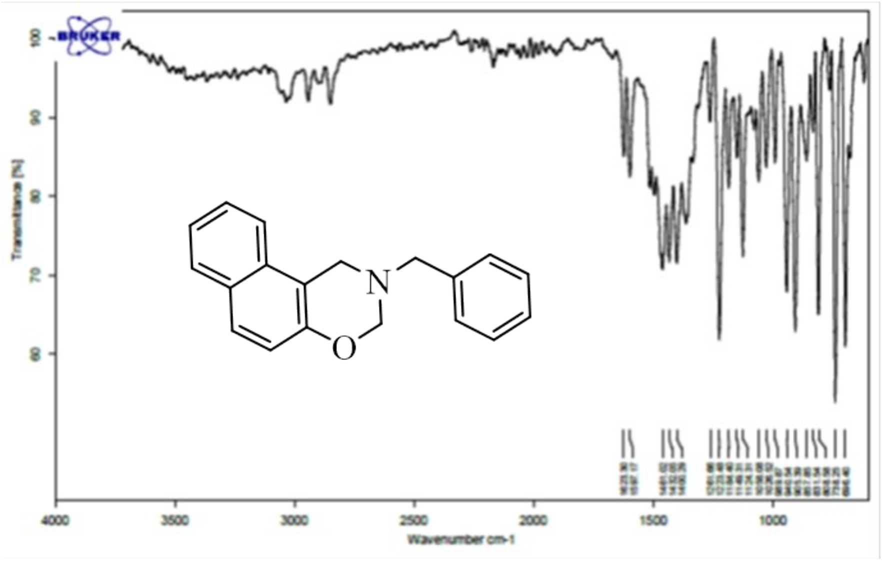

The FT-IR spectrum of product (**4g**)

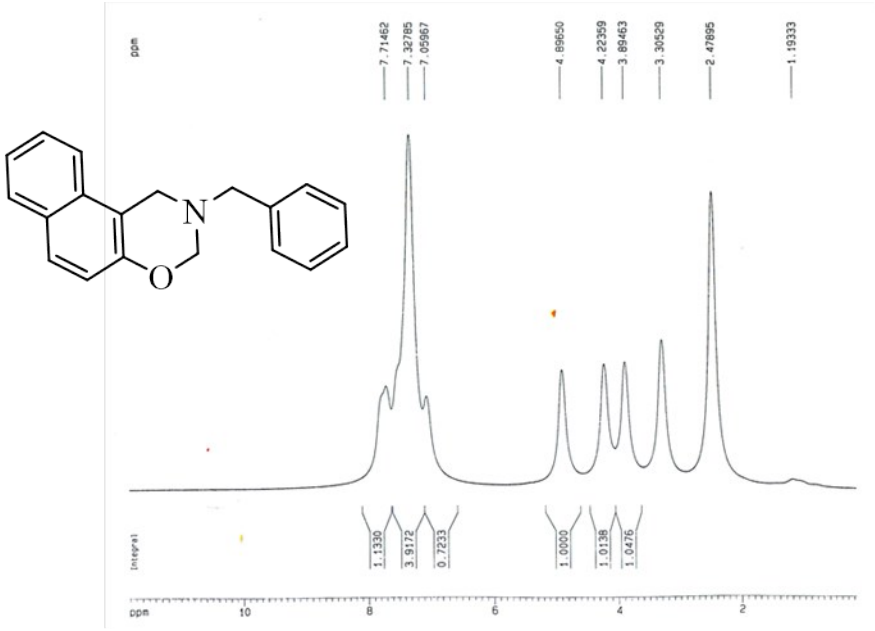

The ^{1}H NMR (500 MHz) spectrum of product (**4g)**

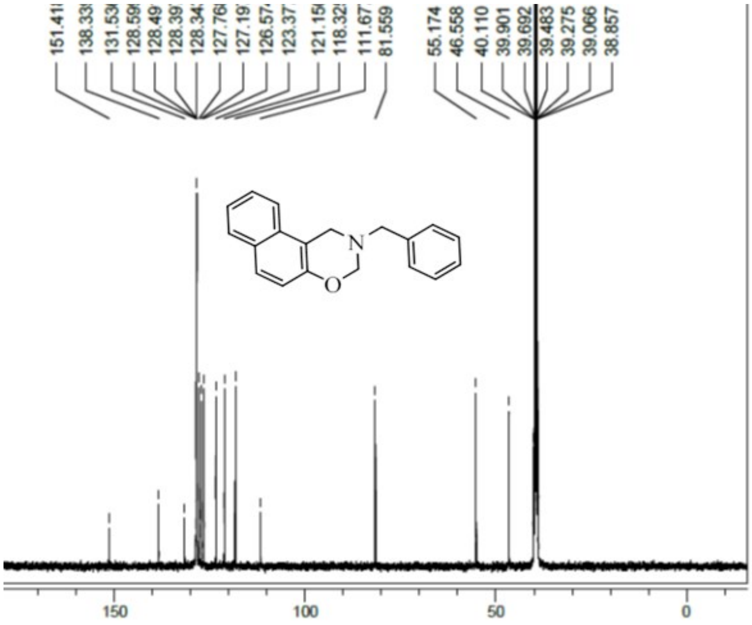

The ^{13}C-NMR (100 MHz) spectrum of product (**4g)**

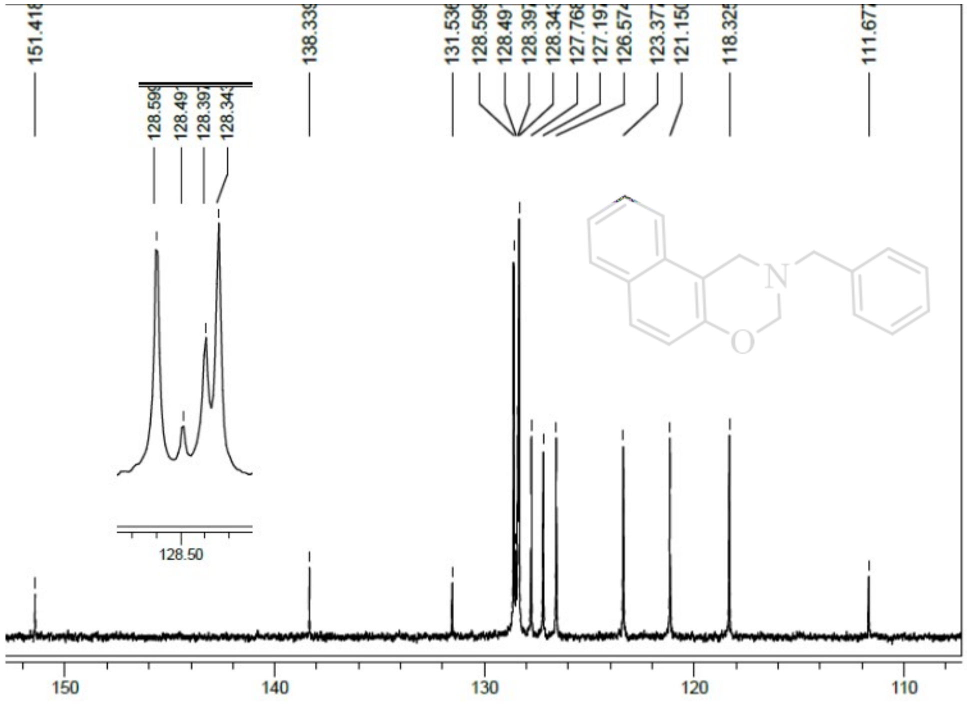

The ^{13}C-NMR (100 MHz) spectrum of product (**4g)**

2-(2-Chlorobenzyl)-2,3-dihydro-1*H*-naphtho[1,2-*e*][1,3]oxazine (4h) ***(Table 2, entry 8):***

Yellow soid, m.p. 70-73 ˚C; FT-IR (ATR) ῡ (cm^{-1}): 1594, 1468, 1226, 1057; ^{1}H NMR (DMSO-d_6, 500 MHz) /δ ppm: 7.07-7.82 (m, 10H, Ar-H), 4.95 (s, 2H, O-CH_2-N), 4.27 (s, 2H, -Ar-CH_2-N), 4.00 (s, 2H, -Ar-CH_2-N); ^{13}C NMR (DMSO-d_6, 100 MHz) /δ ppm: 46.82, 52.56, 81.94, 111.73, 118.33, 121.26, 123.42, 126.46, 127.23, 127.85, 128.39, 128.50, 128.90, 129.35, 130.61, 131.54, 133.23, 135.81, 151.41.

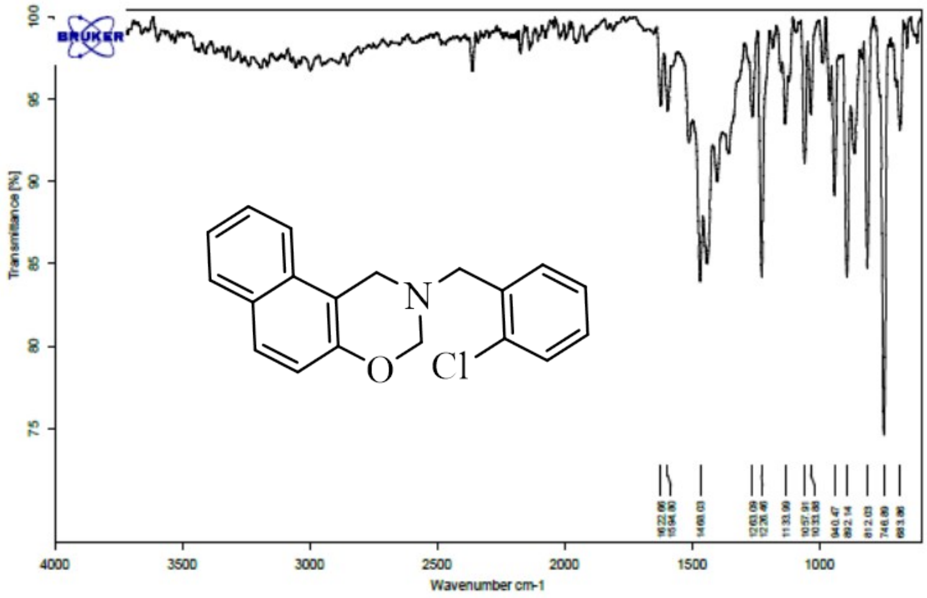

The FT-IR spectrum of product (**4h**)

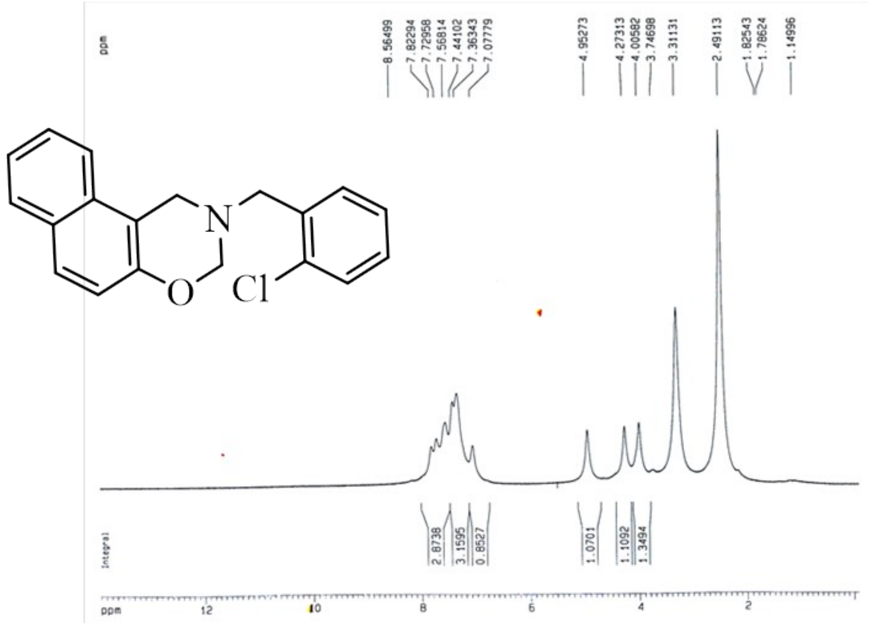

The ^{1}H NMR (500 MHz) spectrum of product (**4h)**

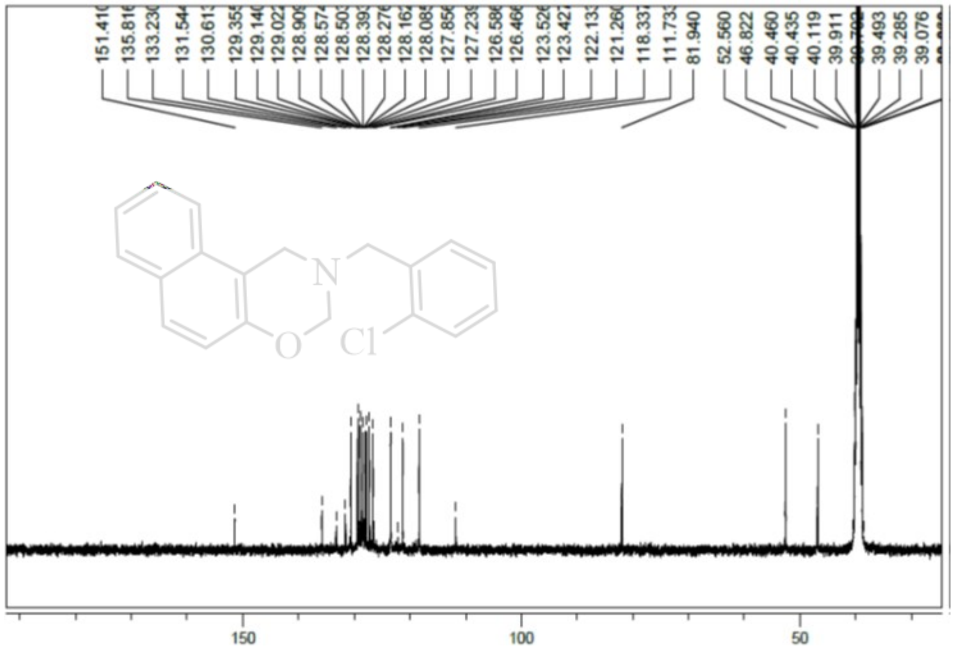

The ¹³C-NMR (100 MHz) spectrum of product (**4h**)

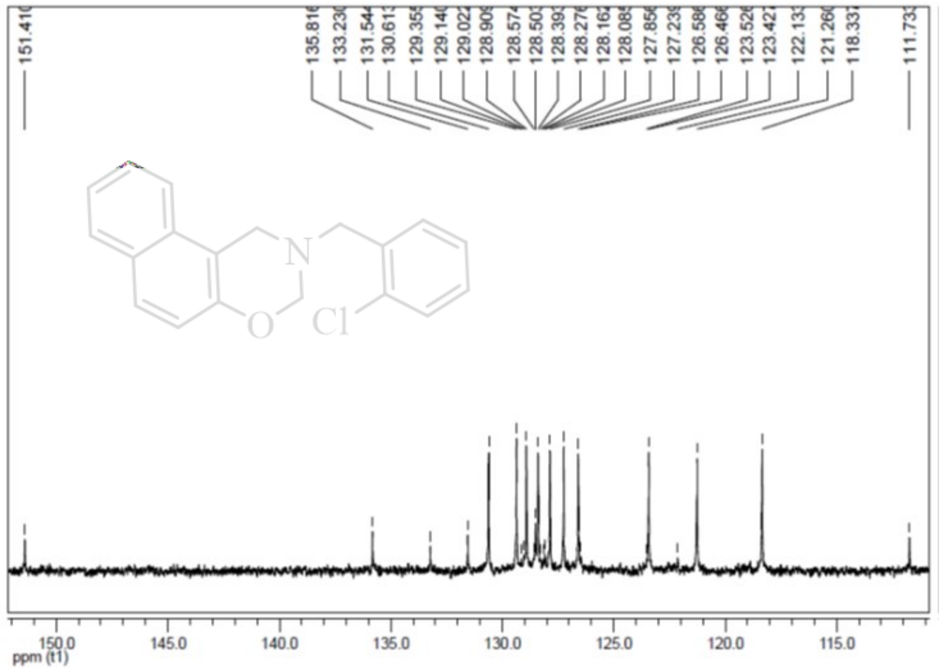

The ¹³C-NMR (100 MHz) spectrum of product (**4h**)

2-Phenethyl-2,3-dihydro-1*H*-naphtho[1,2-*e*][1,3]oxazine(4i) ***(Table 2, entry 9):***

White solid, m.p. 232 °C (d); FT-IR (ATR) ῡ (cm^{-1}): 1597, 1469, 1225, 1060; ^{1}H NMR (DMSO-d$_6$, 500 MHz) /δ ppm: 7.81 (m, 1H, Ar-H), 7.69 (m, 2H, Ar-H), 7.49 (m, 1H, Ar-H), 7.35 (m, 1H, Ar-H), 7.24 (m, 5H, Ar-H), 7.02 (m, 1H, Ar-H), 4.92 (s, 2H, O-CH$_2$-N), 4.32 (s, 2H, -Ar-CH$_2$-N), 2.96 (m, 2H, Ar-CH$_2$-$\underline{CH_2}$-N), 2.88 (m, 2H, Ar-$\underline{CH_2}$-CH$_2$-N); ^{13}C NMR (DMSO-d$_6$, 125 MHz) /δ ppm: 34.86, 47.95, 53.92, 82.69, 113.08, 119.18, 122.26, 124.19, 126.74, 127.33, 128.51, 129.05, 129.22, 129.31, 129.54, 132.43, 140.88, 152.43.

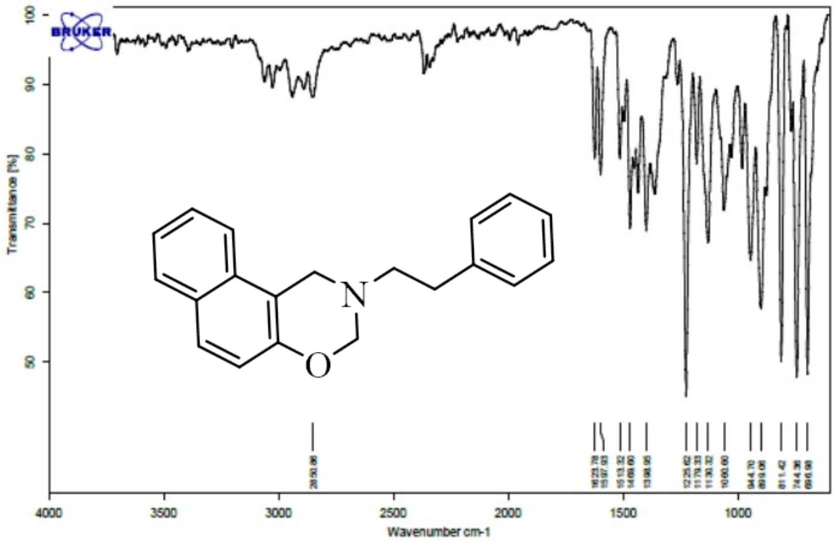

The FT-IR spectrum of product (**4i**)

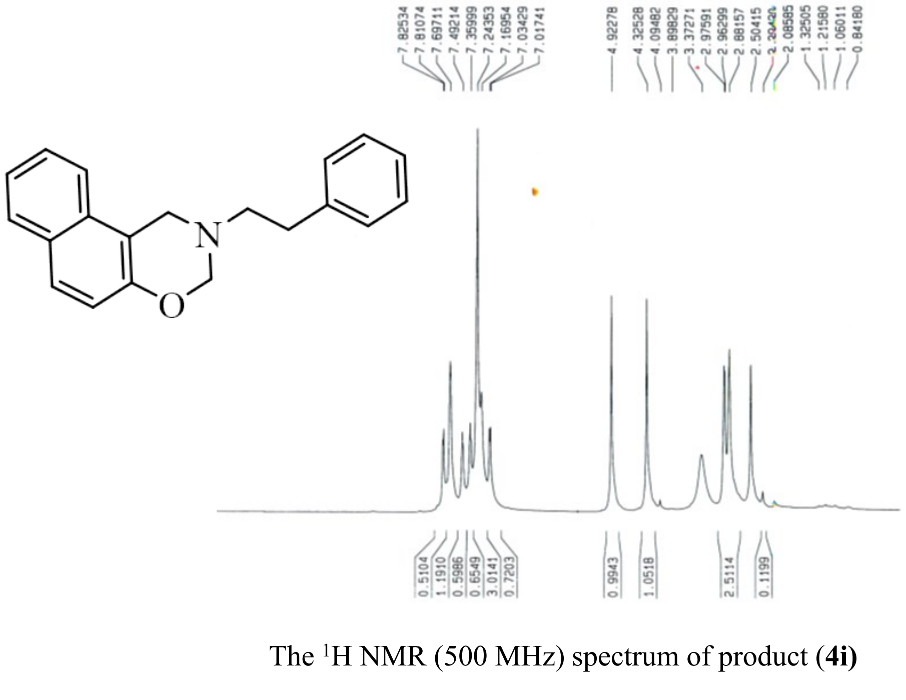

The ^{1}H NMR (500 MHz) spectrum of product (**4i)**

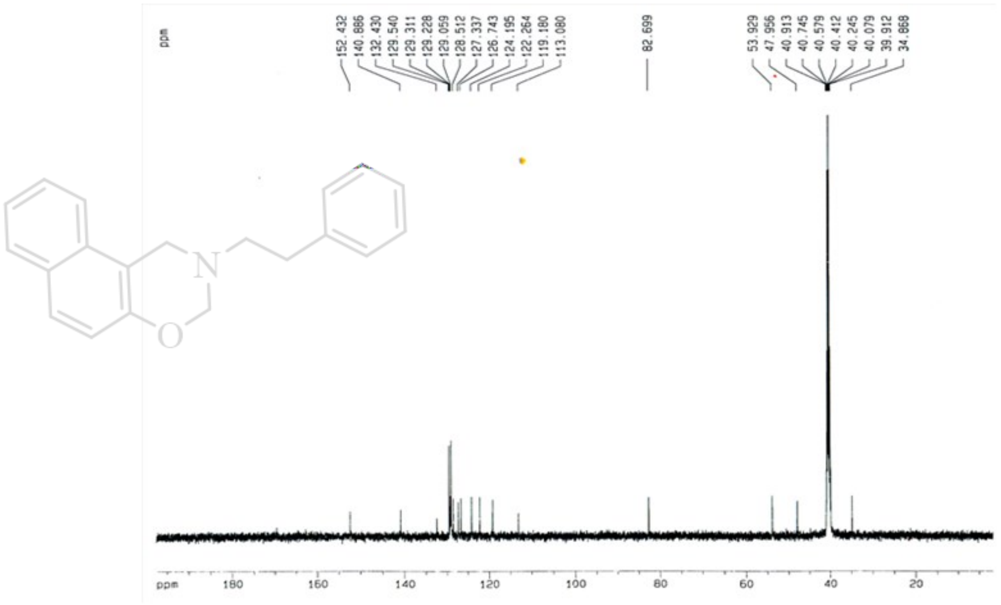

The ^{13}C-NMR (100 MHz) spectrum of product (**4i)**

2-(Furan-2-ylmethyl)-2,3-dihydro-1*H*-naphtho[1,2-*e*][1,3]oxazine(4j) ***(Table 2, entry 10):***

Pale-pink, m.p. 98-100 ˚C; FT-IR (ATR) ῡ (cm^{-1}): 1597, 1467, 1226, 1060; ^{1}H NMR (DMSO-d$_6$, 500 MHz) /δ ppm: 7.71-8.02 (m, 4H, Ar-H), 7.36-7.61 (m, 2H, Ar-H), 7.04 (s, 1H, Ar-H), 6.32-6.52 (m, 2H, Ar-H), 4.89 (s, 2H, O-CH$_2$-N), 4.26 (s, 2H, -Ar-CH$_2$-N), 3.90 (s, 2H, furan-<u>CH$_2$</u>-N); ^{13}C NMR (DMSO-d$_6$, 125 MHz) /δ ppm: 46.37, 47.86, 81.22, 108.76, 110.42, 111.52, 118.27, 121.19, 123.43, 126.60, 127.80, 128.39, 128.50, 131.48, 142.70, 151.29, 151.79.

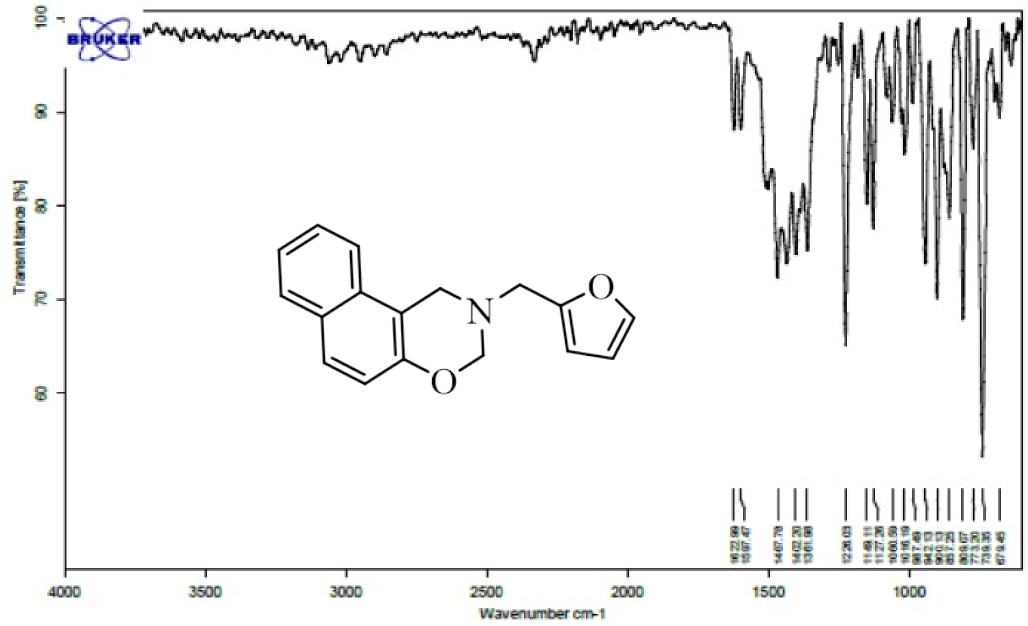

The FT-IR spectrum of product (**4j**)

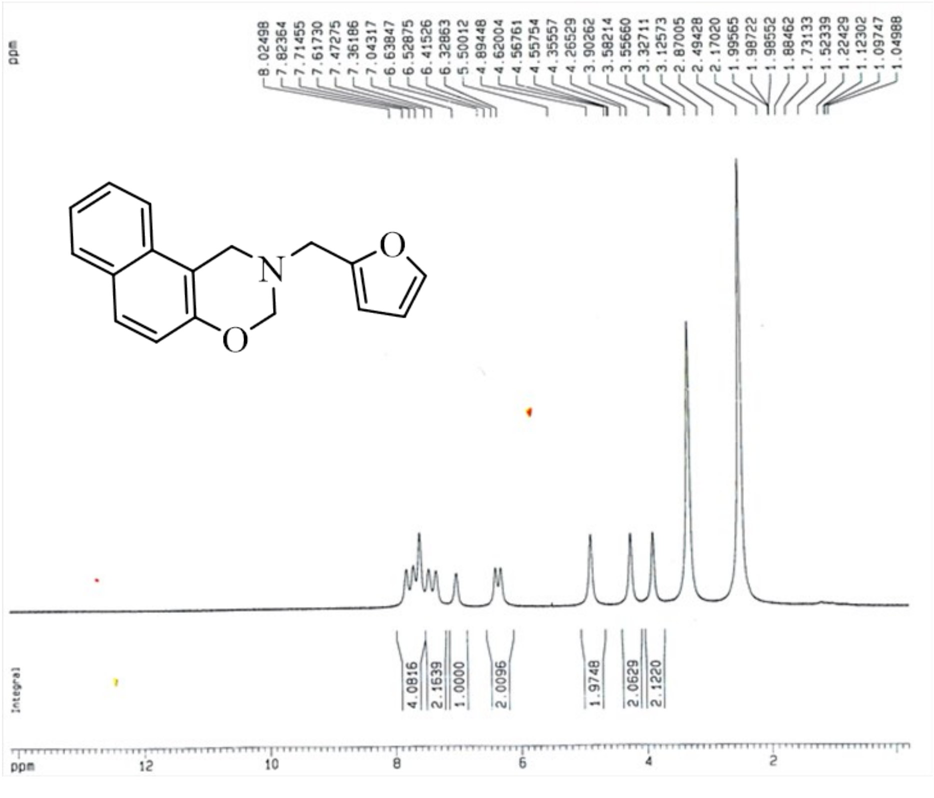

The ^{1}H NMR (500 MHz) spectrum of product (**4j)**

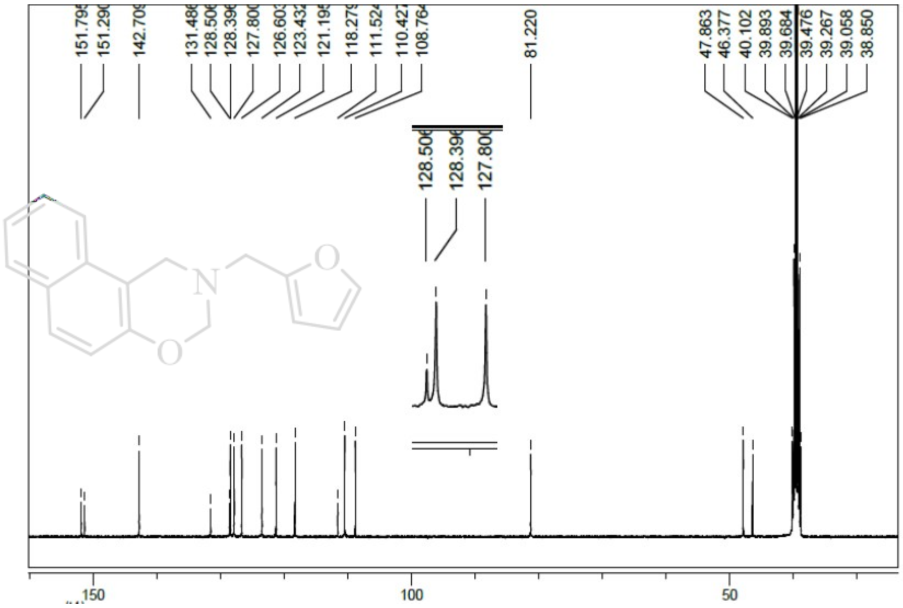

The ^{13}C-NMR (100 MHz) spectrum of product (**4j)**

2-Cyclohexyl-2,3-dihydro-1*H*-naphtho[1,2-*e*][1,3]oxazine(4k) ***(Table 2, entry 11):***

Off-white solid, m.p. 248 ˚C (d); FT-IR (ATR) ῡ (cm^{-1}): 2926, 2852, 1597, 1467, 1227, 1058; ^{1}H NMR (DMSO-d$_6$, 500 MHz) /δ ppm: 7.66-7.81 (m, 3H, Ar-H), 7.48 (m, 1H, Ar-H), 7.35 (m, 1H, Ar-H), 6.98 (m, 1H, Ar-H), 4.99 (s, 2H, O-CH$_2$-N), 4.33 (s, 2H, -Ar-CH$_2$-N), 2.70 (m, 1H, CH-N), 1.08-1.86 (m, 10H, 5CH$_2$).

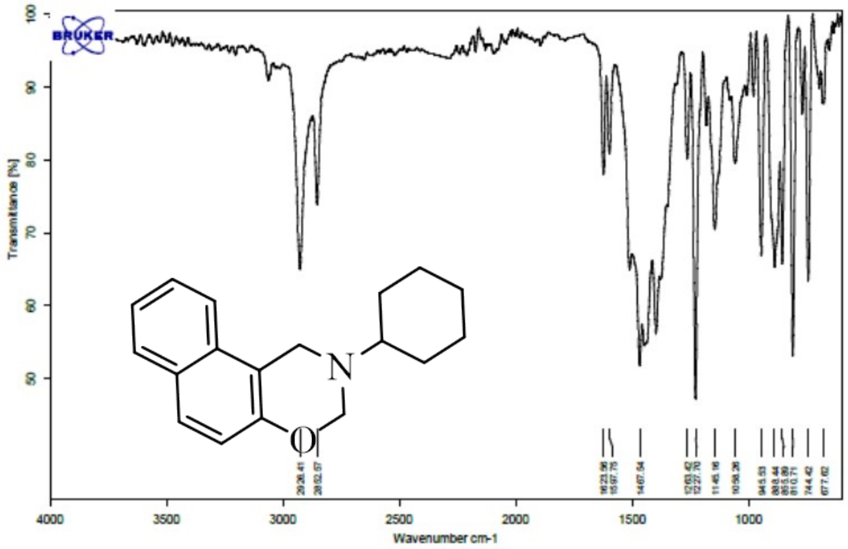

The FT-IR spectrum of product (**4k**)

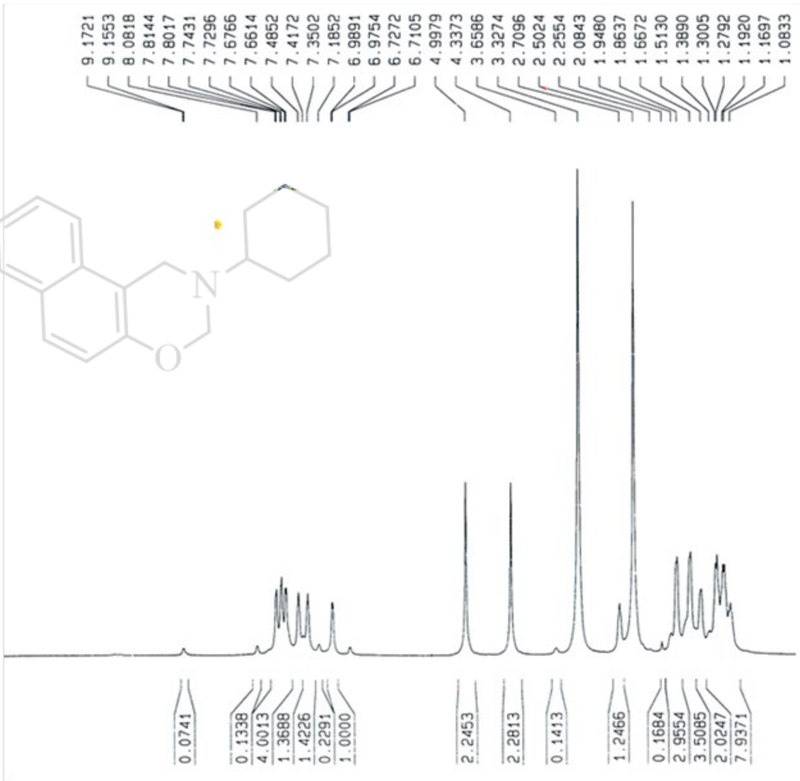

The ^{1}H NMR (500 MHz) spectrum of product (**4k**)

2-Butyl-2,3-dihydro-1*H*-naphtho[1,2-*e*][1,3]oxazine(4l) ***(Table 2, entry 12):***

White solid, m.p. 170 ˚C (d); FT-IR (ATR) ῡ (cm^{-1}): 2954, 2864, 1597, 1433, 1225, 1056.
^{1}H NMR (DMSO-d_6, 500 MHz) /δ ppm: 7.81 (m, 1H, Ar-H), 7.69 (m, 2H, Ar-H), 7.47 (m, 1H, Ar-H), 7.35 (m, 1H, Ar-H), 7.00 (m, 1H, Ar-H), 4.88 (s, 2H, O-CH_2-N), 4.25 (s, 2H, -Ar-CH_2-N), 2.69 (m, 2H, -CH_2-N), 1.53 (m, 2H, CH_2), 1.31 (m, 2H, CH_2), 0.87 (m, 3H, CH_3).
^{13}C NMR (DMSO-d_6, 100 MHz) /δ ppm: 13.80, 19.80, 29.64, 46.94, 50.84, 81.81, 112.08, 118.25, 121.31, 123.26, 126.45, 127.57, 128.32, 128.37, 131.54, 151.51.

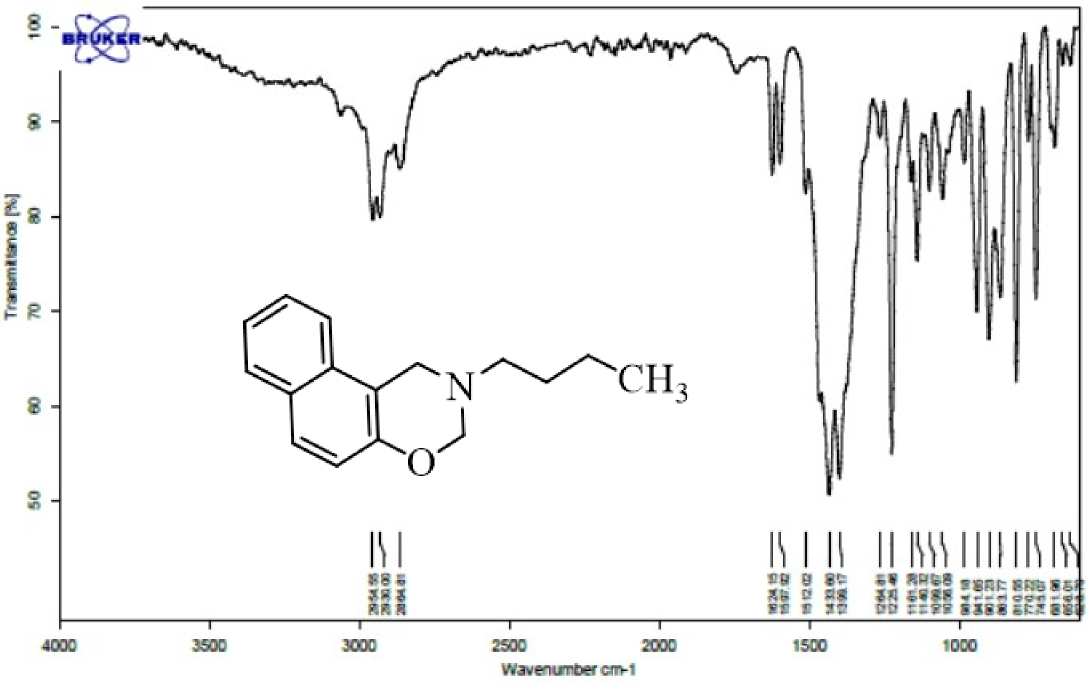

The FT-IR spectrum of product (**4l**)

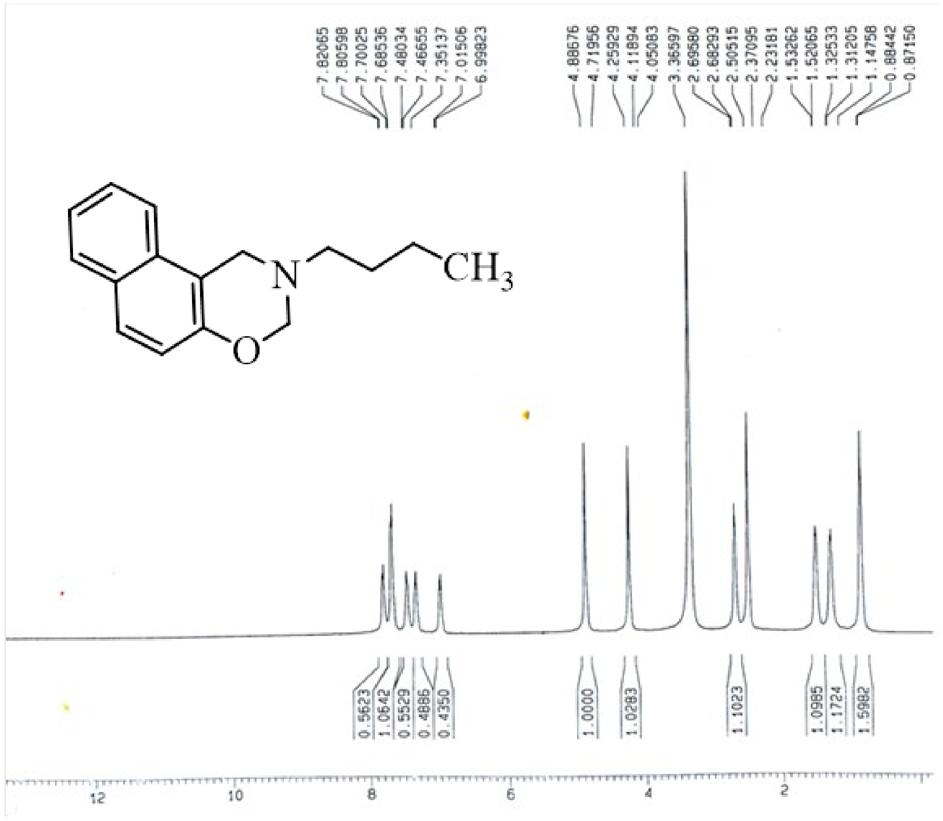

The ^{1}H NMR (500 MHz) spectrum of product (**4l**)

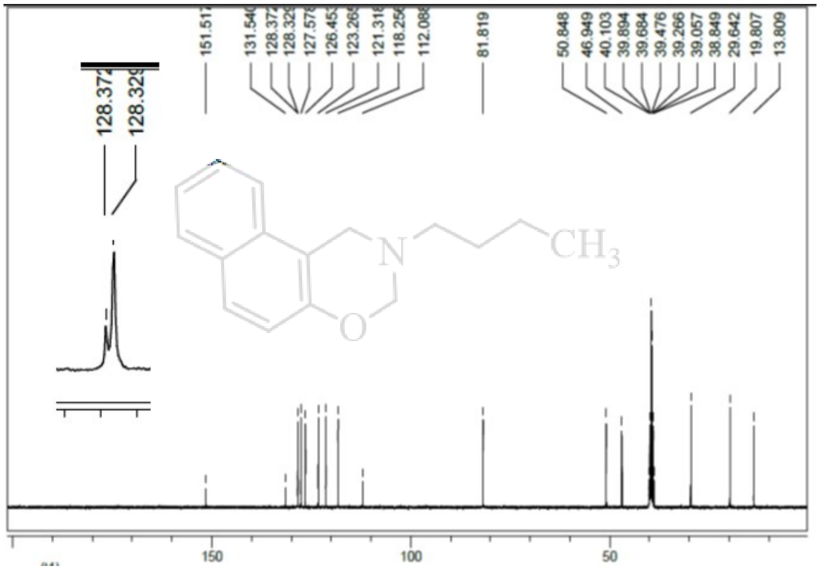

The ^{13}C-NMR (100 MHz) spectrum of product (**4l**)

2-Hexyl-2,3-dihydro-1*H*-naphtho[1,2-*e*][1,3]oxazine(4m) ***(Table 2, entry 13):***

Brown solid, m.p. 177 ˚C (d); FT-IR (ATR) ῡ (cm^{-1}): 2927, 2854, 1597, 1467, 1225, 1057;
^{1}H NMR (DMSO-d$_6$, 500 MHz) /δ ppm: 7.80 (m, 1H, Ar-H), 7.68 (m, 2H, Ar-H), 7.47 (m, 1H, Ar-H), 7.34 (m, 1H, Ar-H), 7.00 (m, 1H, Ar-H), 4.87 (s, 2H, O-CH$_2$-N), 4.25 (s, 2H, -Ar-CH$_2$-N), 2.68 (m, 2H, -CH$_2$-N), 1.53 (m, 2H, CH$_2$), 1.25 (m, 6H, 3CH$_2$), 0.84 (m, 3H, CH$_3$).
^{13}C NMR (DMSO-d$_6$, 125 MHz) /δ ppm: 14.77, 22.98, 27.23, 28.38, 32.02, 47.87, 52.09, 82.71, 112.97, 119.14, 122.18, 124.13, 127.32, 128.46, 129.21, 129.28, 132.44, 152.42.

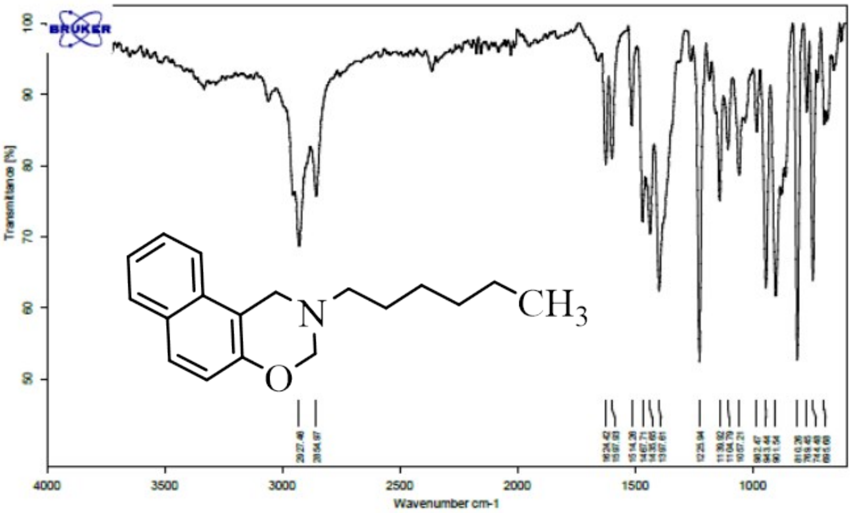

The FT-IR spectrum of product (**4m**)

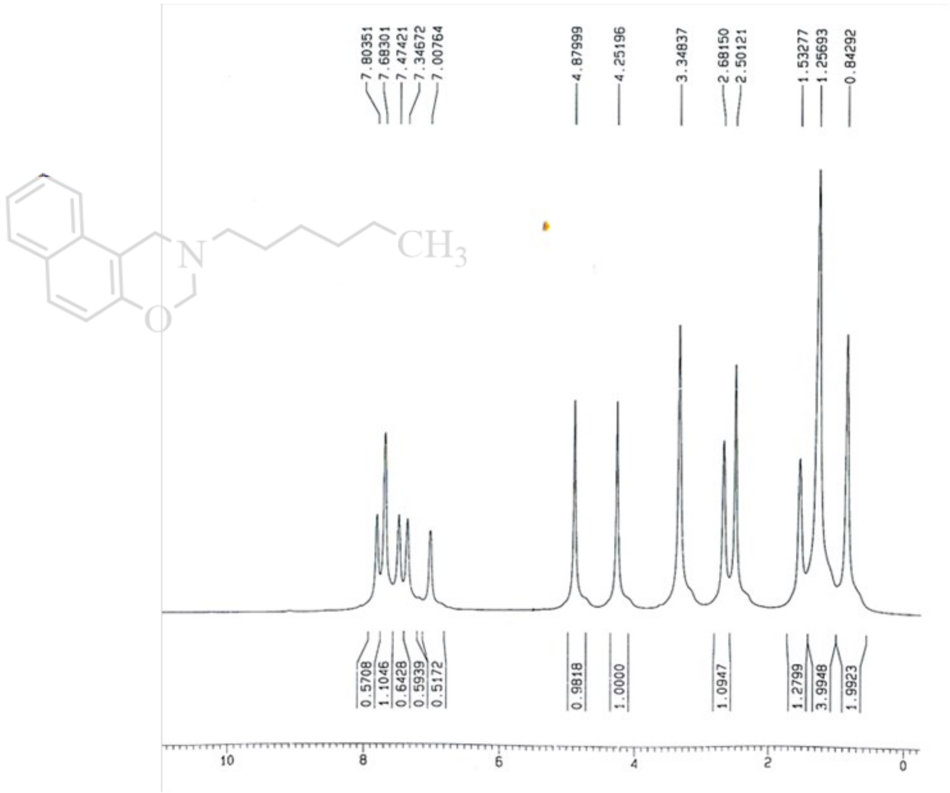

The ^{1}H NMR (500 MHz) spectrum of product (**4m)**

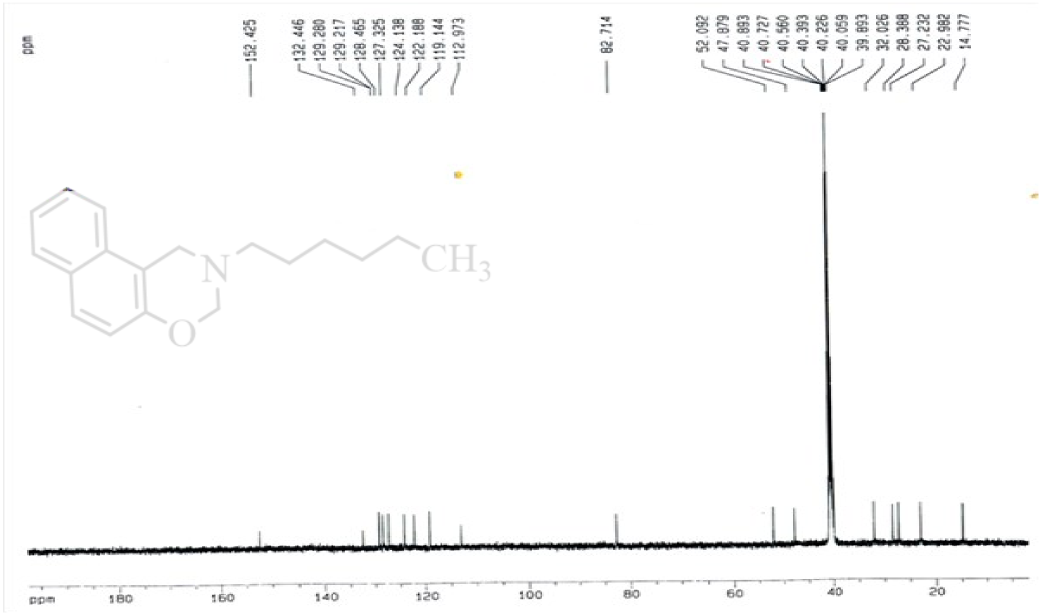

The ^{13}C-NMR (100 MHz) spectrum of product (**4m)**
